# PRDM1 Drives Human Primary T Cell Hyporesponsiveness by Altering the T Cell Transcriptome and Epigenome

**DOI:** 10.3389/fimmu.2022.879501

**Published:** 2022-04-28

**Authors:** Huidong Guo, Ming Wang, Bixia Wang, Liping Guo, Yifei Cheng, Zhidong Wang, Yu-Qian Sun, Yu Wang, Ying-Jun Chang, Xiao-Jun Huang

**Affiliations:** ^1^ Peking University People’s Hospital & Peking University Institute of Hematology, National Clinical Research Center for Hematologic Disease, Beijing Key Laboratory of Hematopoietic Stem Cell Transplantation, Beijing, China; ^2^ Nanfang Hospital, Southern Medical University, Guangzhou, China; ^3^ Peking-Tsinghua Center for Life Sciences, School of Life Sciences, Peking University, Beijing, China; ^4^ Research Unit of Key Technique for Diagnosis and Treatments of Hematologic Malignancies (2019RU029), Chinese Academy of Medical Sciences, Beijing, China

**Keywords:** PRDM1, multiomics, T cell hyporesponsiveness, HSCT, GvHD

## Abstract

T cell hyporesponsiveness is crucial for the functional immune system and prevents the damage induced by alloreactive T cells in autoimmune pathology and transplantation. Here, we found low expression of *PRDM1* in T cells from donor and recipients both related to the occurrence of acute graft-versus-host disease (aGVHD). Our systematic multiomics analysis found that the transcription factor PRDM1 acts as a master regulator during inducing human primary T cell hyporesponsiveness. *PRDM1*-overexpression in primary T cells expanded Treg cell subset and increased the expression level of *FOXP3*, while decreased expression had the opposite effects. Moreover, the binding motifs of key T cell function regulators, such as FOS, JUN and AP-1, were enriched in PRDM1 binding sites and that PRDM1 altered the chromatin accessibility of these regions. Multiomics analysis showed that PRDM1 directly upregulated T cell inhibitory genes such as *KLF2* and *KLRD1* and downregulated the T cell activation gene *IL2*, indicating that PRDM1 could promote a tolerant transcriptional profile. Further analysis showed that PRDM1 upregulated *FOXP3* expression level directly by binding to *FOXP3* upstream enhancer region and indirectly by upregulating KLF2. These results indicated that PRDM1 is sufficient for inducing human primary T cell hyporesponsiveness by transcriptomic and epigenetic manners.

## Introduction

T cell hyporesponsiveness protects individuals from immune-related pathologies such as autoimmune diseases and complications of transplantation. Graft-versus-host disease (GVHD), a disordered immune system pathology induced by alloreactive T cells, remains an obstacle to the success of allogeneic hematopoietic stem cell transplantation (allo-HSCT). Several T cell inhibitory signals have been demonstrated to play a role in GVHD mouse models, such as PRDM1, and a lack of *Prdm1* was found to shorten the life span of GVHD mice (1). However, the role of PRDM1 in human primary T cells and its relationship with GVHD occurrence in patients after allo-HSCT have not been elucidated.

PRDM1, also known as B lymphocyte-induced maturation protein-1 (BLIMP-1), was initially identified as an essential regulator of B lymphocyte development and plasma cell terminal differentiation. Studies have shown that mice with B cell-specific deletion of *Prdm1* lack a defined plasma cell compartment and have severely reduced serum immunoglobulin titers (2, 3). *Prdm1* also governs B cell fate decision-related transcription factors (TFs), such as MYC, PAX5 and CIITA, establishes a transcriptional program during B lymphocyte differentiation and is considered to be a master regulator of terminal B cell differentiation (4–6). Subsequent studies revealed PRDM1’s essential role in maintaining T cell self-tolerance and homeostasis in mouse models with T cell-specific deletion of *Prdm1*. Mice deficient in *Prdm1* showed an accumulation of activated T cells and developed multiorgan inflammatory disease (7, 8). Moreover, *Prdm1* was found to maintain the function and homeostasis of Treg cells by cooperating with interferon regulatory factor 4 (IRF4) or preventing methylation of conserved noncoding sequence 2 (CNS2) in the *Foxp3* region (9, 10). These studies explored the phenotypic and functional roles of PRDM1 in maintaining T cell tolerance. However, systemic analyses to define the PRDM1 regulatory network in T cell hyporesponsiveness are still needed.

Although sophisticated transgenic mouse models provide adequate cells and are a useful platform for investigating biological processes regulated by TFs, there are still disparities between mouse models and humans. As a benefit of the advances in high-throughput sequencing technology, it is possible to investigate the precise regulatory network of TFs from human primary cells with low input cell numbers. These technologies can help us restore the regulatory landscape in humans. Assay for transposase-accessible chromatin with sequencing (ATAC-seq) has been widely used to assess chromatin accessibility during biological processes due to its advantages of capturing native chromatin states and requiring a low number of cells (11, 12). Cleavage under targets and tagmentation (CUT&Tag), similar to chromatin immunoprecipitation with sequencing (ChIP-seq) but requiring much lower input cell numbers, provides a snapshot of *in situ* TF interactions with chromatin elements (13, 14).

In this study, our data showed the essential role of PRDM1 in regulating primary T cell hyporesponsiveness. Low *PRDM1* expression level in T cells from donors or recipients correlated with high aGVHD occurrence after allo-HSCT. Comprehensive epigenetic and transcriptional analyses identified that PRDM1 altered the chromatin accessibility of key T cell TF-targeted regions and promoted tolerant transcription profiling in human primary T cells. Furthermore, our data showed that PRDM1 upregulated FOXP3 in human T cells by directly binding to upstream enhancers and indirectly by upregulating KLF2. Taken together, our study demonstrated that PRDM1 is sufficient for inducing human primary T cell tolerance.

## Materials and Methods

### Samples

**Figure 1:**
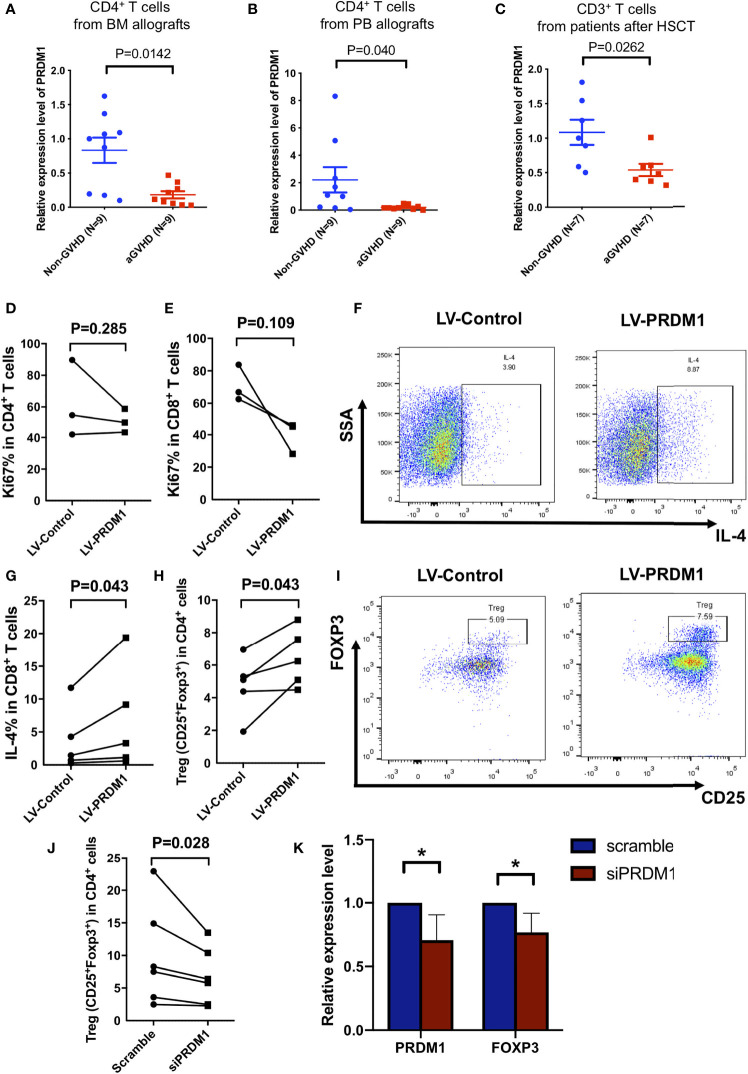
High expression level of *PRDM1* suppressed human primary T cell function. **(A, B)** Quantitative real-time PCR showed the expression level of *PRDM1* in CD4^+^ T cells from bone marrow allografts **(A)** and from peripheral allografts **(B)** and the aGVHD occurrence in related patients, respectively. **(C)** The expression level of *PRDM1* in peripheral CD3^+^ T cells in patients with aGVHD (n = 7) after allo-HSCT compared with those without aGVHD (n = 7) in the same period. Mann–Whitney U test was used for testing *PRDM1* expression. **(D, E)** Flow cytometry results showed the percentage of Ki67 expression level on *PRDM1* overexpressing CD4^+^ T cells **(D)** and CD8^+^ T cells **(E)** in GFP^+^ population compared with control T cells (n = 3). **(F, G)** Representative flow cytometry results and percentages showed the IL-4 secretion level of *PRDM1* overexpressing GFP^+^CD8^+^ T cells compared with control T cells (n = 5). **(H, I)** Percentages and representative flow cytometry results showed the Treg cell subset (CD4^+^CD25^+^FOXP3^+^) in *PRDM1* overexpressing GFP^+^CD3^+^ T cells compared with control T cells (n = 5). **(J)** Percentage of Treg cell subset in *PRDM1* knocked down T cells compared with control T cells (n = 6). **(K)** Quantitative real-time PCR was used to detect *PRDM1* and *FOXP3* expression level in *PRDM1* knocked down T cells compared with control T cells (n = 6). Wilcoxon rank sum test was performed to assess the significance in D-K. *P < 0.05.

Peripheral blood was collected from healthy donors or patients undergoing allo-HSCT. Peripheral grafts and bone marrow grafts were collected from 18 healthy donors between 1 November 2018 and 31 December 2018, and the related allo-HSCT patients were followed up to 30 October 2020 (**Table S1**, related to **Figures 1A, B** and **Figures S1A,B**). Bone marrow was collected from healthy donors. Peripheral blood was collected from 14 patients with or without acute GVHD (aGVHD) after allo-HSCT (**Table S2**, related to **Figure 1C**). Peripheral blood was collected from 13 patients with or without acute GVHD (aGVHD) after allo-HSCT (**Table S3**, related to **Figure S3**). 100-day aGVHD and chronic GVHD (cGVHD) were diagnosed and graded according to the standard international criteria (15, 16). Human bone marrow mononuclear cells (BMMCs) or peripheral blood mononuclear cells (PBMCs) were isolated by Ficoll density centrifugation. The study has been approved by the Ethics Committee of Peking University People’s Hospital, and written informed consent from all subjects was obtained in accordance with the Declaration of Helsinki.

### Overexpression *PRDM1* in Primary T Cells

CD3^+^ T cells were purified from BMMCs by CD3 microbeads (Miltenyi Biotec, 130-097-043) according to manufacturer’s instructions. CD4 conventional T cells (CD4^+^CD25^-^) were isolated by negative selection with CD25 microbeads (Miltenyi Biotec, 130-092-983) followed by positive selection with CD4 microbeads (Miltenyi Biotec, 130-045-101). T cells were pre-stimulated with Dynabeads Human T-Activator CD3/CD28 beads (Invitrogen, 11131D) and 100 U/ml rhIL-2 in IMDM medium (Gibco, Invitrogen) containing 10% BIT 9500 (Stemcell Technologies, 09500) for 24 h. The *PRDM1*-overexpressing lentivirus was purchased from Sangon Biotech (Shanghai, China). After 24 h, the cells were transduced with lentiviruses and added polybrene (Sigma, USA) at a dose of 6 μg/ml, then incubated for another 24 h at 37°C and 5% CO_2_. The cells were incubated for another 24 h at 37°C and 5% CO_2,_ and fresh medium was changed. GFP^+^ cells were isolated after a 72 h infection and cultured in IMDM containing 10% BIT 9500 with 100 U/ml rhIL-2 routinely used.

### siRNA Transfection

5×10^5^ CD3^+^ T cells from Granulocyte colony stimulating factor (G-CSF) mobilized healthy donors were stimulated with CD3/CD28 beads in IMDM medium containing 10% BIT 9500 with rhIL-2 for 24 h. Then the cells transfected with Smartpool-On-TARGET plus PRDM1 siRNA (Dharmacon, L-009322-00-0005) or ON-TARGET plus Non-targeting control Pool (Dharmacon, D-001810-10-05) with lipo3000 (Invitrogen, L3000015) according to a previously published article (17). Cells were analyzed after total 72 h transfection.

### Flow Cytometry

Surface staining was performed at room temperature (RT) with antibodies for 20 min. Cells were stained with surface antibody to human CD4-Percp-cy-5.5, CD8-APC-R700/V500, or CD25-APC (BD Pharmingen) for 20 min at RT. For cytokine detection, T cells were stimulated with phorbol 12-myristate 13-acetate (PMA, 50 ng/mL, Sigma Aldrich, 880134P) and ionomycin (1000 ng/mL, Sigma Aldrich, I3909) for 4 h at 37°C in the presence of GolgiPlug (BD Pharmingen, 555029). Intracellular staining of IL-4-APC, FOXP3-PE or Ki67-PE was performed by using the Transcription Factor Buffer Set kit (BD Pharmingen, 562574) according to the manufacturer’s instructions, and then incubated for 20 min with antibodies at RT. Detailed antibody information was listed in **Table S4**.

### Quantitative Real-Time PCR

RNA was extracted with RNeasy mini kit (QIAGEN, 74106) according to the manufacturer’s protocol. Amount of RNA was quantified with Qubit (ThermoFisher). Synthesis of cDNA was carried out with cDNA reverse transcription kit (TaKaRa, RR047A). Q-PCR was performed with SYBR Green (Roche, 04913914001). A 7500 real-time PCR system (Applied Biosystems) was used to detect the signal. Values were normalized according to the expression of the housekeeping gene *18S* in the same samples by using a 2^-ΔΔCT^ method. The primer sequences were listed in the **Table S5**.

### Western Blot

5×10^6^ Jurkat cells were resuspended and lysed in 100ul SDS-PAGE loading buffer (Beyotime, Shanghai, China, P0015L). Protein from these samples electrophoresed on 10% SDS-PAGE gel and transferred to polyvinylidene fluoride membranes. Membranes were blocked with 5% skimmed milk powder in TBST (Tris Buffered saline Tween) for about 1 h and incubated overnight with primary antibodies at 4°C, followed by secondary antibodies for 1 h. In western blot analysis, PRDM1 (1:200), FOXP3 (1:1000), β-ACTIN (1:1000) and KLF2 (1:1000) were used for immunoblotting with secondary antibodies conjugated with horseradish peroxidase. Detailed information about the antibodies was listed in **Table S4**.

### RNA-Sequencing

GFP^+^ cells from LV-PRDM1 groups or LV-Control groups were isolated by flow cytometric sorting. RNA was extracted using the RNeasy Micro Kit (QIAGEN, 74004) and purified by NEBNext Oligo d(T)_25_ beads (NEB, E7490). NEBNext Ultra II RNA Library Prep Kit (NEB, E7770S) was used to generate cDNA libraries. AMPure XP beads (Beckman Coulter, A63881) were used to purified cDNA libraries between 300 and 500bp. Paired-end sequencing was performed on a NovaSeq6000 (Illumina), producing between 28 and 35 million 150-bp pair-end reads per sample.

The quality of the raw fastq files was examined with the FastQC software. Raw fastq files were trimmed using the software Trimmomatic by setting the parameter “LEADING: 3 TRAILING: 3 SLIDINGWINDOW: 4:15 MINLEN: 36”. The trimmed fastq files were then aligned to the human GRC38/hg38 reference genome using Hisat2, and gene expression was quantified by StringTie (18). Then differential expression analysis was run using DESeq2 package. GO enrichment analysis was performed using DAVID (https://david.ncifcrf.gov/). GSEA was performed using the Broad Institute software (http://software.broadinstitute.org/gsea/index.jsp). Enrichment scores were calculated by comparing LV-PRDM1 to LV-Control groups.

### CUT&Tag

1×10^5^ Flow cytometric sorted PRDM1 overexpressed CD3^+^ T cells were performed CUT&Tag using Hyperactive *In-Situ* ChIP Library Prep Kit (Vazyme, TD901) as previously described (13). Briefly, aliquots of cells were washed twice in Wash Buffer then incubated with activated Concanavalin A coated magnetic beads for 10 min at RT. 1:20 diluted PRDM1 primary antibody or IgG antibody was incubated overnight with samples on a rotating platform at 4°C. Anti-rabbit secondary antibody was diluted 1:100 and incubated at RT for 60 min on a rotating platform. Cells were washed using the magnet to remove unbound antibodies. Hyperactive pG-Tn5 adapter complex with a concentration of 0.04 μM was added to the cells with gentle vortexing and incubated at RT for 1 h. Next, cells were incubated with Tagmentation buffer at 37°C for 1 h to fragment. Then DNA barcoded with TruePrep Index Kit V2 for Illumina (Vazyme, TD202) and used for amplifying libraries. VAHTS DNA Clean Beads (Vazyme, N411) were used to purified cDNA.

Paired-end reads were aligned to hg38 reference genome using Bowtie2 (13). Picard tool was used to remove presumed PCR duplicates using the MarkDuplicates command. Bam files containing uniquely mapped reads were created using Samtools. Bigwig files were converted from Bam files by the bamCoverage command of deepTools (https://deeptools.readthedocs.io/en/develop/content/tools/bamCoverage.html). Peaks were called using MACS2 with q value cutoff of 1e+05, -f BAMPE and IgG as control. Genes proximal to peaks were annotated against the hg38 genome using Bioconductor package ChIPpeakAnno. PRDM1 binding motifs were identified using findMotifsGenome.pl from HOMER.

### ATAC-Sequencing

ATAC-sequencing was performed according to previous described (11). Briefly, GFP^+^ cells from LV-PRDM1 groups or LV- Control groups were isolated by flow cytometric sorting. 5×10^4^ sorted cells resuspended and lysed in 50 μl Lysis Buffer (10 mM NaCl,10 mM Tris-HCl, pH 7.4, 3 mM MgCl_2_, 0.1% ICEPALEA-630) after twice wash in PBS. DNA was fragmented using TruePrep DNA Library Prep Kit V2 for Illumina (Vazyme, TD501-01) and purified by using MinElute PCR Purification Kit (QIAGEN, 28006). Purified DNA barcoded with TruePrep Index Kit V2 for Illumina (Vazyme, TD202) and amplified by PCR using TruePrep DNA Library Prep Kit V2 for Illumina. VAHTS DNA Clean Beads (Vazyme, N411) were used to purified cDNA libraries between 100 and 1000bp. Paired-end sequencing was performed on a NovaSeq6000 (Illumina).

Raw ATAC-seq fastq files from paired-end sequencing were processed according to a previous described (19). Clean fastq files were aligned to the hg38 reference genome using Bowtie2. Samtools was used to remove unmapped, unpaired, mitochondrial reads. PCR duplicates were removed using Picard. Reads were shifted +4bp and -5bp for positive and negative strand respectively. Peak calling was performed using MACS2 with FDR q-value 0.01. We combined peaks of all samples to create a union peak list and merged overlapping peaks with BedTools merge command. The number of reads in each peak was determined using BedTools coverage. Differentially accessible regions were identified following DESeq2 normalization using an FDR cut-off q value < 0.05. Motif enrichment was calculated using HOMER (default parameters) on peaks differentially accessible across LV-PRDM1 group and LV-Control group. Transcription binding site prediction analysis was performed using known motif discovery strategy.

### Methylation Analysis

Bisulfite-converted DNA from *PRDM1* overexpressed T cells or control T cells was extracted by using DNA micro kit (QIAGEN, 74004) and transformed by using EZ DNA Methylation-Gold kit (ZYMO RESEARCH, D5006) according to manufacturer’s instructions. Illumina BEADLAB SYSTEM was use for analyzing MethylationEPIC BeadChip array. For pyrosequencing, methylated regions were amplified by PCR containing 10 ng of bisulfite-treated DNA, 10 μM forward and reverse primers, EpiTap PCR Buffer, 25 mM MgCl_2_, 2.5 mM dNTP mixture and 5 U/μl EpiTap HS in a final volume of 50 μl reaction according to the manufacturer’s instructions (TaKaRa R110A). Amplification and sequencing primers were according to a published article (20). Amp4: r-AACCCTCAAACCTAACTCATAC; q-GGAGGTGATAGTAAAGAAAGGA; Amp5: p-TGTTTGGGGGTAGAGGATTT; o-TATCACCCCACCTAAACCAA.

### KLF2 Inhibition

Human primary CD3^+^ T cells were infected with *PRDM1*-overexpressing lentivirus as above described, GFP^+^ T cells were sorted using flow cytometry and cultured in complete medium and treated with either vehicle (methanol) or 10 μM geranylgeranyl pyrophosphate (GGPP; Sigma, G6025) (21). Cells were collected and analyzed by qPCR after 8 h cultured.

### Statistical Analyses

All the results are shown as mean ± SEM. Wilcoxon rank sum test was used to assess difference in paired cytometric data and mRNA expression. Mann–Whitney U test was used for testing unpaired mRNA expression. When comparing more than two groups, non-parametric techniques were used, which was first analyzed by Friedman Test then analyzed by Wilcoxon rank sum test. *P values < 0.05 were considered to be significant. Statistical analyses were performed on SPSS 20.0 software.

## Results

### PRDM1 was Sufficient for Inducing Human Primary T Cell Hyporesponsiveness

We first analyzed relationship between the expression level of *PRDM1* in donor-derived T cells and the occurrence of GVHD after allo-HSCT. We detected the expression level of *PRDM1* in CD4^+^ T cells and CD8^+^ T cells from bone marrow (BM) allografts or peripheral blood (PB) allografts and monitored for the occurrence of GVHD after HSCT for 2 years. Low expression levels of *PRDM1* in both CD4^+^ T cells and CD8^+^ T cells from BM or PB grafts correlated with aGVHD in patients after allo-HSCT but not in those without aGVHD, and the results in CD4^+^ T cells from BM or PB grafts were statistically significant (**Figures 1A, B** and **Figures S1A, B**, **Table S1**). Moreover, T cells from patients with aGVHD (n = 7) showed lower *PRDM1* expression levels than those without aGVHD in the same period after HSCT (n = 7) (**Figure 1C**, **Table S2**). These results suggested that high expression of *PRDM1* in T cells might be related to T cell tolerance in GVHD patients. To investigate whether PRDM1 inhibited T cell activation in human primary T cells, we overexpressed *PRDM1* in CD3^+^ T cells from healthy donors with lentivirus. T cells were purified from healthy donors and stimulated with CD3/CD28 beads, then infected with control GFP expressing lentivirus or *PRDM1*-overexpressing lentivirus (**Figures S1C, D**). qPCR analysis showed that *PRDM1* expression level was significantly increased in the *PRDM1* overexpression group (LV-PRDM1) compared with that in the control group (LV-Control) (**Figure S2A**). Overexpressed *PRDM1* in CD3^+^ T cells did not change the proportion of CD4^+^ T cells, CD8^+^ T cells or the ratio of CD4/CD8 in human primary T cells (**Figures S2B–D**). A trend toward inhibition of CD8^+^ T cell proliferation was observed in *PRDM1* overexpressed group (**Figures 1D, E**). IL-4 secretion level was increased in CD4^+^ and CD8^+^ T cells from *PRDM1*-overexpession group compared with that control group (**Figures 1F, G** and **Figure S2E**). CD4^+^CD25^+^FOXP3^+^ Treg cell subset expansion was also observed in the LV-PRDM1 group compared with the LV-Control group (**Figures 1H, I**). To further investigate whether Treg expansion or induction of FOXP3 expression contributes to the Treg cells increasing in *PRDM1* overexpressed T cells, firstly we analyzed the Ki67 level in Treg cells. The results showed that the proliferation ability had no difference between Treg cells in PRDM1 overexpressing group compared with control group (**Figure S2F**). Then we overexpressed *PRDM1* in CD4^+^CD25^-^ T cells from healthy donors with lentivirus. CD4^+^CD25^-^ T cells were isolated by negative selection with CD25 microbeads followed by positive selection with CD4 microbeads. Then CD4^+^CD25^-^ T cells were stimulated with CD3/CD28 beads and infected with control GFP expressing lentivirus or *PRDM1*-overexpressing lentivirus. Flow cytometry analysis showed that Treg (CD4^+^CD25^+^FOXP3^+^) cells were increased in *PRDM1* overexpressing group compared with control group (**Figures S2G, H**). The protein level of FOXP3 was also increased in CD4^+^CD25^+^ subsets from *PRDM1* overexpressing group compared with control group (**Figure S2I, J**), which is indicated that Treg cells increased in *PRDM1* overexpressed T cells mainly due to high expression level of *PRDM1* induced FOXP3 expression level. Moreover, we detected Treg cell population and FOXP3 protein levels in aGVHD and non-GVHD patients by flow cytometry. The results showed that the ratio of Treg cells was decreased in aGVHD patients compared with non-GVHD patients (**Figures S3A, B**, **Table S3**). The protein level of FOXP3 was also decreased in CD4^+^CD25^+^ T cells from aGVHD patients compared with non-GVHD patients (**Figures S3C, D**, **Table S3**). These results indicated that low *PRDM1* expression level might corelated with low FOXP3 protein level and decreased Treg cells in patients with aGVHD. Taken together, our results indicated high expression level of *PRDM1* in human primary T cells promoted T cells into hyporesponsiveness state.

Our previous study analyzed the transcriptome of G-CSF-mobilized T cells and found upregulated expression levels of *PRDM1* (22). We validated this result *via* qPCR, and the *PRDM1* expression level was upregulated in G-CSF-mobilized T cells from healthy donors (**Figure S4A**). To verify the necessity of PRDM1 for T cell tolerance, we knocked down the expression level of *PRDM1* in G-CSF-mobilized T cells. Consistent with the previous results, inhibiting the *PRDM1* expression level decreased the ratio of Treg cell subset (**Figure S4B**, **Figure 1J**). However, the IL-4 secretion level in CD4^+^ T cells or CD8^+^ T cells showed no difference in PRDM1 knockdown group compared with that scramble group (**Figure S4C**). The expression level of *FOXP3* was decreased after *PRDM1* knockdown (**Figure 1K**). These data demonstrated that high expression level of *PRDM1* inhibited T cell activation and induced hyporesponsiveness in human primary T cells.

### T Cells Highly Expressing *PRDM1* Exhibited Tolerance-Related Transcription Profiles

**Figure 2:**
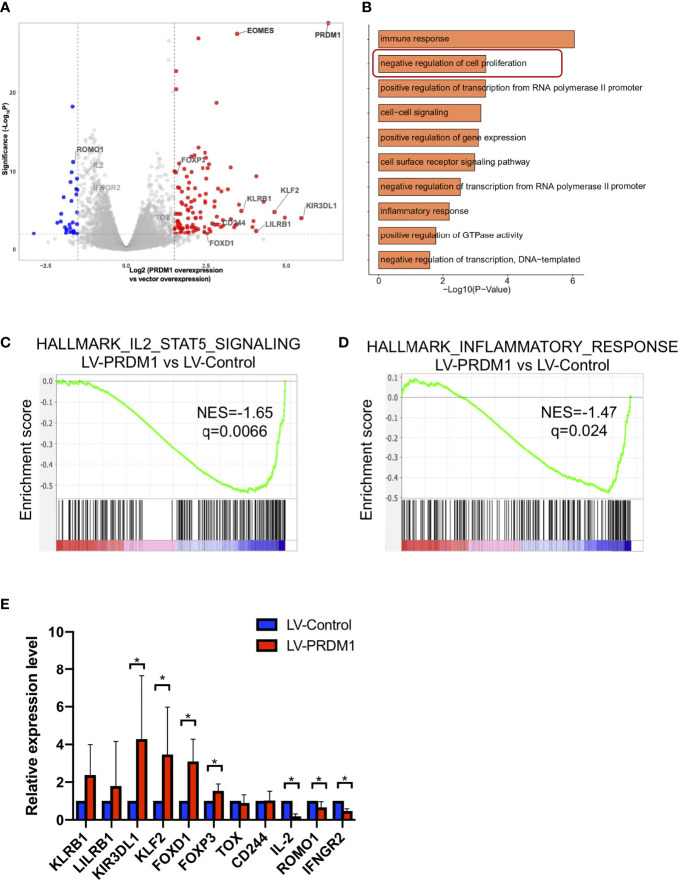
T cell highly expressing *PRDM1* exhibited tolerant transcription profiling. **(A)** RNA-seq volcano plots comparing gene expression between *PRDM1*-overexpressing T cells and control T cells (n = 3). FDR q-value < 0.01, |log_2_fold change| > 1.5. **(B)** Gene pathway enrichment analysis for the upregulated genes in *PRDM1*-overexpressing T cells. **(C, D)** GSEA pathway enrichment plot in *PRDM1*-overexpressing T cells versus control T cells. NESs and P values are shown for each gene set. P values were calculated by Kolmogorov-Smirnov test. **(E)** Quantitative real-time PCR was used to validate upregulated and downregulated genes in *PRDM1*-overexpressing T cells detected by RNA-seq (n = 5). Wilcoxon rank sum test was performed to assess the significance of relative gene expression. *P < 0.05.

Due to the long coding region of PRDM1, the infection efficiency in primary T cells is very low (**Figures S1C, D**), it is difficult to acquire a sufficient number of *PRDM1*-overexpression CD4^+^ or CD8^+^ T cells for further multiomics sequencing. Considering that overexpressed *PRDM1* in human primary T cells did not alter the proportion of CD4^+^ and CD8^+^ T cells, we performed multiomics sequencing on sorted infected bulk CD3^+^ T cells (GFP^+^). RNA-seq analysis showed that several negative regulators of T cell function like *EOMES* (1), *KLF2* (23), *LILRB1* (24), *KLRB1* (25), and *CD244* (also known as *2B4*) (26), were upregulated in the *PRDM1*-overexpression group compared with the control group (**Figure 2A**). GO analysis showed that there was enrichment of the negative regulation of the cell proliferation signaling pathway in the *PRDM1*-overexpression group (**Figure 2B**). Analysis of pathway activity revealed that *PRDM1* overexpression resulted in a global reduction in IL-2 and inflammatory response signaling pathways, suggesting a major impact on the global state of T cells (**Figures 2C, D**). We further validated the RNA-seq results *via* qPCR. A similar expression profile was observed in the LV-PRDM1 group (**Figure 2E**). These results demonstrated that high expression level of *PRDM1* promoted human primary T cells exhibiting tolerance-related transcription profiles.

### PRDM1 Mediated Transcriptional Regulation of T Cell Tolerance

**Figure 3:**
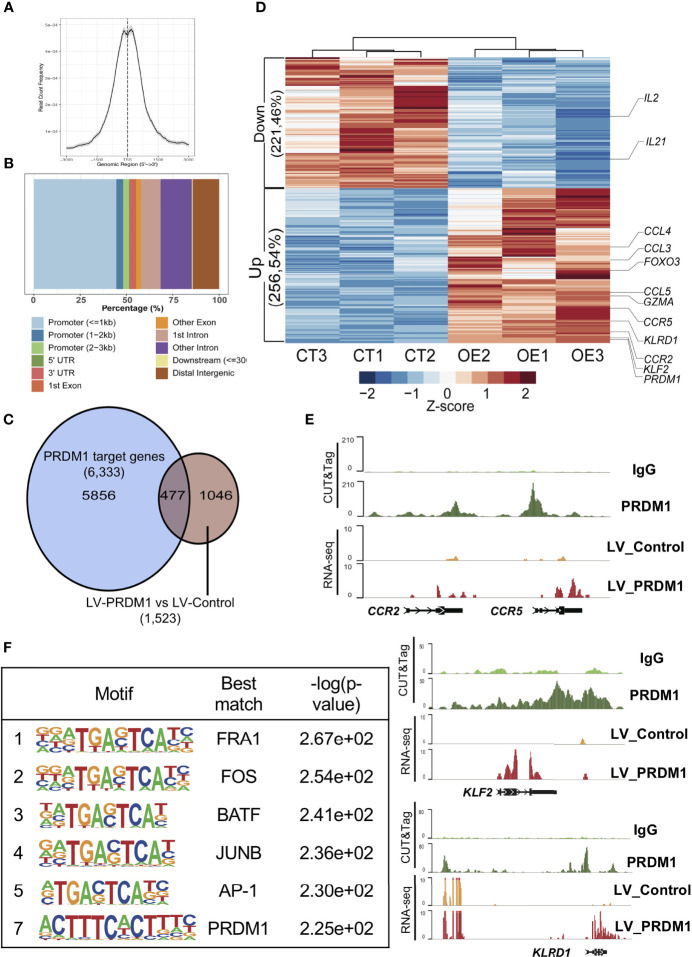
PRDM1 directly upregulated T cell inhibitory gene expression in primary T cells. **(A)** Aggregate plot of PRDM1 binding site around transcription start sites. **(B)** Locations of PRDM1 binding sites. **(C)** The overlap between PRDM1 target genes and differential expression genes in *PRDM1*-overexpressing T cells compared with control T cells. **(D)** Heatmap showed PRDM1 directly regulated differential expression genes in *PRDM1*-overexpressing T cells (OE group) compared with control T cells (CT group). FDR q-value < 0.05, |log_2_fold change| > 1. **(E)** WashU browser views showed PRDM1 biding sites (CUT&Tag) and related gene expression level (RNA-seq). **(F)** Top 7 enriched transcription factor motifs in the PRDM1 CUT&Tag peaks are shown.

Previous results showed that *PRDM1* overexpression elevated the expression of several T cell hyporesponsiveness-related genes. To further investigate the mechanism of PRDM1 regulating T cell transcriptome, we conducted CUT&Tag assessment of PRDM1 to define its target genes. We investigated the abundance of PRDM1 binding sites around transcription start sites (TSSs) and found that the majority of detected PRDM1 binding sites were enriched at TSSs (**Figure 3A** and **Figure S5A**). Nearly 50% of PRDM1 binding sites were located in promoter regions (**Figure 3B** and **Figure S5B**). Combined analysis of CUT&Tag and transcriptome data (RNA-seq) revealed that 477 genes were directly bound and regulated by PRDM1 (**Figure 3C**). Among these genes, several T cell inhibitory genes were directly upregulated by PRDM1, such as *KLRD1* (also known as CD94) (27, 28) (**Figures 3D, E**). T cell tolerance-maintaining genes like *KLF2* (23) were also upregulated by PRDM1 (**Figures 3D, E**). A previous study showed that loss of expression of chemokines such as *CCL3*, *CCL4* and *CCR5* was correlated with T cell migration and activation in autoimmune mouse models (29). We also found that PRDM1 directly upregulated *CCL3*, *CCL4* and *CCR5* in human primary T cells and might contribute to T cell hyporesponsiveness in *PRDM1*-overexpressing T cells (**Figures 3D E**). Consistent with a previous study in a mouse model (30), PRDM1 directly downregulated *IL2* gene expression levels in human primary T cells (**Figure 3D**). Analyzing the DNA binding motifs in the PRDM1 CUT&Tag data revealed the expected PRDM1 motif (**Figure 3F**). Moreover, binding sites for several key T cell regulation-related TFs, such as FRA1, FOS, BATF, JUNB and AP-1, were also significantly enriched in locations in which PRDM1 bound (**Figure 3F**), suggesting that PRDM1 might act as a master regulator during T cell hyporesponsiveness.

### PRDM1 Remodeled the Chromatin Accessibility of Key T Cell Tolerance Regulation-Related Target Regions

**Figure 4:**
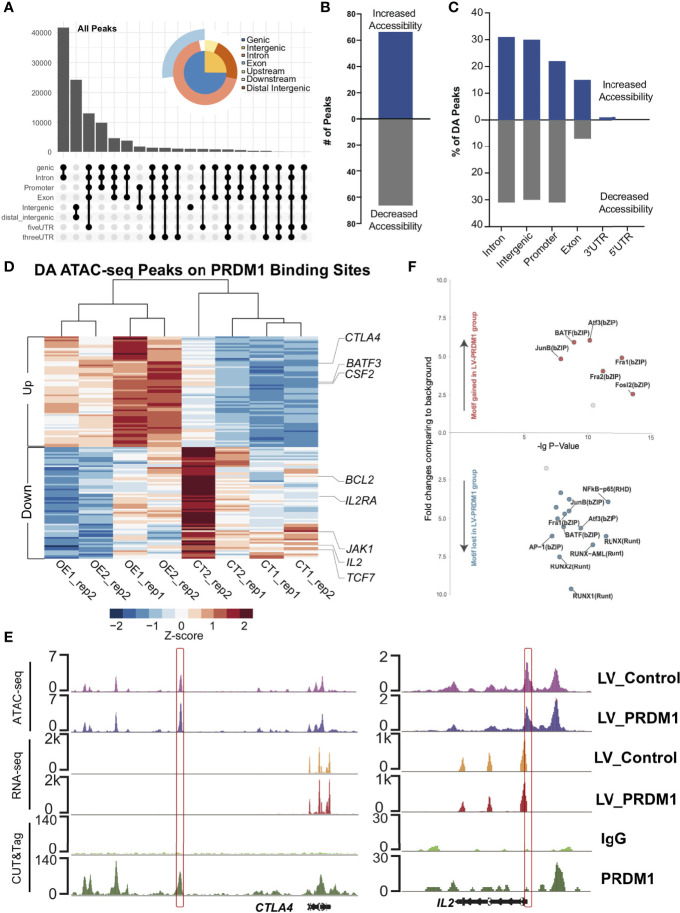
PRDM1 reshaped the epigenetic profile of T cell tolerance regulation-related target regions. **(A)** Categories of *cis*-element in all open chromatin regions (OCRs). **(B)** Overall OCR peak changes for *PRDM1*-overexpressing T cells compared to control T cells. **(C)** Categories of *cis*-element OCR peaks that changed between *PRDM1*-overexpressing T cells and control T cells. **(D)** Heatmap shown differentially accessible (DA) peaks overlapped with the PRDM1 CUT&Tag binding peaks between *PRDM1*-overexpressing T cells and control T cells. Selected genes assigned to the peaks are indicated. FDR q-value < 0.05. **(E)** WashU browser views showed PRDM1 biding sites (CUT&Tag), related gene expression level (RNA-seq) and chromatin accessibility (ATAC-seq). **(F)** Transcription factor motif gain or loss associated with overexpression of PRDM1. X axis represents the -logP value of the motif enrichment. Y axis represents the fold change of the motif enrichment. Targeted motifs in the changed OCR between the LV-PRDM1 and LV-Control groups were compared to the whole genome background to calculate p value and fold change.

As we observed that the binding sites of PRDM1 enriched several key T cell regulator motifs, we hypothesis that PRDM1 might act as a core factor during T cell hyporesponsiveness process. Therefore, we performed ATAC-seq on *PRDM1*-overexpressing primary T cells and control T cells. Most open chromatin regions (OCRs) showed enrichment of gene body regions (**Figure 4A** and **Figure S5C–E**). Although there were abundant OCRs in both *PRDM1*-overexpressing T cells and control T cells, fewer OCRs were different between the control and *PRDM1* overexpression groups, with equal numbers of peaks gained or lost (**Figures 4A, B**). Most of the changes were located in intronic, intergenic and promoter regions (**Figure 4C**). We next assigned each OCR to the nearest gene to identify genes that could be regulated by these cis-regulatory elements. We found that most of differentially accessible (DA) region proximal genes in *PRDM1*-overexprssion T cells were PRDM1 target genes (77%, **Figures 4D** and **Figure S5F**). For example, *BATF3* showed gained chromatin accessibility in the *PRDM1* overexpression group, which might be related to the increased IL-4 secretion in *PRDM1*-overexpressing T cells (31, 32) (**Figure 4D**). The T cell inhibitory gene *CTLA4* showed gained chromatin accessibility in the *PRDM1* overexpression group, consistent with the hyporesponsive phenotype of T cells after *PRDM1* overexpression (**Figures 4D, E**). In contrast, *IL2RA* and *IL2* showed decreased chromatin accessibly in the *PRDM1* overexpression group, consistent with the RNA-seq and CUT&Tag data (**Figures 4D, E**), which indicated that PRDM1 bound to the *IL2* locus and remodeled the chromatin structure during regulation of the IL-2 signaling pathway. Next, we identified the TF motifs present in the OCRs that were dependent on PRDM1 for altered accessibility. Interestingly, there were a high number of overlapping TF motifs between the changed OCR-enriched TF motifs and the PRDM1 binding site-enriched TF motifs, such as FRA1, BATF, JUNB and AP-1 (**Figures 3F**, **Figure 4F**). This result suggested that PRDM1 altered chromatin accessibility when it bound to target sites. Because these regions are the binding sites of TFs that play a central role in regulating T cell function, PRDM1 might act as a core TF in regulating T cell hyporesponsiveness.

### PRDM1 Induced *FOXP3* Expression by Directly Binding to Upstream Enhancers and Indirectly by Upregulating KLF2

**Figure 5:**
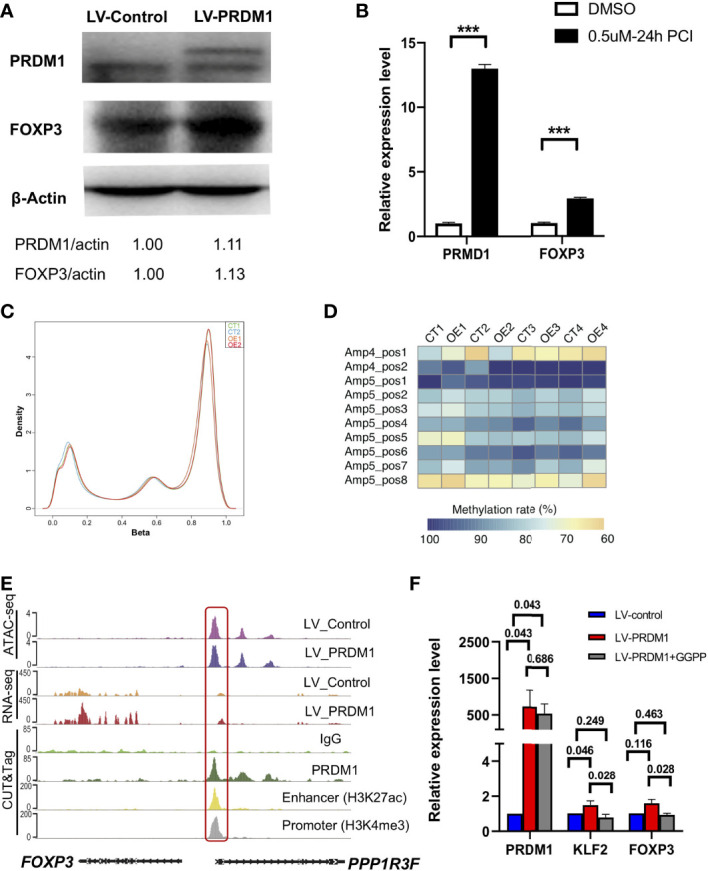
PRDM1 induced *FOXP3* expression by directly binding to upstream enhancer and indirectly by upregulating KLF2. **(A)** Western blot showed the protein level of PRDM1 and FOXP3 in *PRDM1* overexpressing Jurkat T cells compared with control T cells (two experiment repeats). **(B)** Quantitative real-time PCR showed the expression level of *PRDM1* and *FOXP3* in Jurkat cells treated with DMSO or PCI-24781. **(C)** Density of beta values distribution in *PRDM1*-overexpressing T cells and control T cells. **(D)** Heatmap showed the degree of methylation at each CpG motif according to the color code. **(E)** WashU browser views showed PRDM1 biding sites and histone modifications around *FOXP3* gene locus. **(F)** Quantitative real-time PCR showed the expression level of *PRDM1*, *KLF2* and *FOXP3* in control T cells, *PRDM1*-overexpressing T cells and KLF2 inhibitor GGPP treated *PRDM1*-overexpressing T cells, respectively (n = 6). Friedman Test showed the P value is 0.03 for *PRDM1*, *KLF2* and *FOXP3*. Then P value was analyzed by Wilcoxon rank sum test. ***P < 0.05.

Our previous results showed that *FOXP3* was coexpressed with *PRDM1* in human primary T cells, which is *FOXP3* expression level was increased in *PRDM1* overexpressed T cells and *FOXP3* expression level was decreased in *PRDM1* knockdown T cells (**Figure 2A, E** and **1J**). To further verify the coexpression relationship of FOXP3 with PRDM1, we overexpressed *PRDM1* in the Jurkat cell line and performed western blotting. The results also showed increased mRNA and protein levels of FOXP3 in the LV-PRDM1 group compared with the LV-Control group (**Figure 5A** and **Figures S6A–C**). Considering that Jurkat cells are a tumor cell line and might have different characteristics from primary T cells, we analyzed the phenotype of Jurkat cells overexpressing *PRDM1*. The results showed that a high expression level of *PRDM1* inhibited the proliferation of Jurkat cells and arrested Jurkat cells in the G1 phase (**Figures S6D, E**). qPCR results showed downregulation of the cell cycle-activating gene *CCND2* (**Figure S6F**). Pan-histone deacetylase (HDAC) inhibitors have been reported to elevate *PRDM1* expression levels in follicular lymphoma cells (33). Here, we employed PCI-24781 (abexinostat), a novel HDAC inhibitor, to treat Jurkat cells and found that PCI-24781 inhibited Jurkat proliferation and elevated mRNA and protein expression levels of both PRDM1 and FOXP3 (**Figure 5B** and **Figures S6G–I**). These results indicated that PRDM1 inhibited T cell function in both human primary T cells and leukemia T cells. Moreover, FOXP3 was coexpressed with PRDM1 in human T cells.

Previous studies have reported that *Prdm1* deficiency in mouse Treg cells leads to heavy methylation of CNS2 in the *Foxp3* locus and a loss of *Foxp3* expression (10). To investigate the mechanism underlying the elevated FOXP3 mRNA and protein levels in *PRDM1*-overexpressing T cells, we analyzed the genomic methylation level in *PRDM1*-overexpressing primary T cells with the MethylationEPIC BeadChip array. Unsupervised clustering showed that overexpression of *PRDM1* did not change the methylation level of the genome of primary T cells (**Figures S7A–C**). The generated density plot and boxplot showed that there were no significant changes in the distribution of the beta values in *PRDM1*-overexpressing T cells (OE group) compared with the control group (CT) (**Figure 5C**). To further investigate whether PRDM1 changes the methylation level of the *FOXP3* locus, we employed pyrosequencing to identify the specific methylation pattern of the *FOXP3* gene region. According to a previous study, we chose amplicon 4 (Amp4) and amplicon 5 (Amp5) according to previous study in T cells (20), these regions also include the CNS2 region of the *FOXP3* gene (**Figure S7D**). The results showed that there were similar methylation patterns in the Amp4 and Amp5 regions between *PRDM1*-overexpressing T cells and control T cells (**Figure 5D**). These results demonstrated that PRDM1 did not upregulate FOXP3 by changing the methylation pattern and that the regulatory mechanism by which PRDM1 affects FOXP3 in human T cells may be different from that in the mouse model.

To further investigate the mechanism underlying PRDM1-induced FOXP3 expression, we analyzed the PRDM1 binding region around the *FOXP3* locus. PRDM1 did not bind to the *FOXP3* gene coding region but bound upstream of the *FOXP3* locus (**Figure 5E**). We integrated histone modification data (CUT&Tag data of the enhancer marker H3K27ac and the promoter marker H3K4me3 in primary T cells) from our previous study (22). Interestingly, the PRDM1 binding site around the *FOXP3* locus was in the enhancer region, which is also the promoter region of *PPP1R3F* (**Figure 5E**). *PPPIR3F* is a protein-coding gene; however, it is barely expressed in T cells. Our RNA-seq data also showed a low expression level of *PPPIR3F* in *PRDM1*-overexpressing T cells and control T cells (**Figure 5E**). These results indicated that PRDM1 might directly activate *FOXP3* expression level by bring upstream enhancer into physical proximity with *FOXP3* promoter. Previous studies have shown that the TF KLF2 stabilizes *Foxp3* expression levels and enhances Treg cell maintenance of peripheral tolerance in mouse models (23, 34). We also observed that PRDM1 directly upregulated *KLF2* based on RNA-seq and CUT&Tag data (**Figure 3E**). To determine whether KLF2 is involved in *FOXP3* upregulation in *PRDM1*-overexpressing T cells, we used the KLF2 inhibitor GGPP to treat *PRDM1*-overexpressing human primary T cells. The results showed that *PRDM1* overexpression elevated both *KLF2* and *FOXP3* expression levels, while the KLF2 inhibitor downregulated both *KLF2* and *FOXP3* expression levels (**Figure 5F**). This result demonstrated that KLF2 also involved in PRDM1 regulating *FOXP3* expression level. Taken together, these results indicated there might exist distinct mechanism of PRDM1 upregulating *FOXP3* expression level, which is directly binding to *FOXP3* upstream enhancer and indirectly by upregulating KLF2.

## Discussion

Here, we systematically report the central role of PRDM1 in inducing human primary T cell hyporesponsiveness. Overexpression of *PRDM1* in human primary T cells induced Treg cell expansion and increased IL-4 secretion. Inhibiting *PRDM1* expression decreased the ratio of Treg cells. Moreover, the transcriptional profile of *PRDM1*-overexpressing T cells exhibited tolerance-related signatures. Integrated multiomics analysis demonstrated that PRDM1 altered the chromatin accessibility of PRDM1 binding sites, which are also binding sites of many key regulators of T cell function. We also found that PRDM1 directly upregulated FOXP3 by binding to the upstream enhancer region of the *FOXP3* locus and indirectly upregulated KLF2.

The essential role of *Prdm1* in maintaining T cell homeostasis and self-tolerance has been well established by conditional knockout mouse models. Studies have shown that *Prdm1* controls the later stages of T cell maturation and homeostasis (35–37). Loss of PRDM1 function led to an accumulation of effector T cells and severe multiorgan inflammation (7, 8). PRDM1 was found to directly bind to the *Il2* and *Fos* genes to inhibit the production of IL-2 and attenuated the proliferation of activated T cells (30, 38). Although accumulated evidence indicates the essential role of *Prdm1* in maintaining T cell tolerance, the global regulatory model of PRDM1 in T cell tolerance has not been revealed. We took advantage of multiomics technology and systematically analyzed the transcriptional and epigenetic profiles of *PRDM1*-overexpressing human T cells. Previous study identified PRDM1 inhibited IL-2 in mouse model (30), we also observed inactive IL-2 in *PRDM1* overexpressing human primary T cells by integrating analysis of RNA-seq, ATAC-seq and CUT&Tag data. *Xin et al.* found that in *Prdm1* ^-/-^ T cells, chemotaxis and adhesion molecules (*Ccl3*, *Ccl9*) and effector marker (*Gzma*, *Klrg1*) were downregulated (39). Here in our study, we also found chemotaxis and adhesion related genes including *CCL3*, *CCL4*, *CCL5*, *CCR2* and *CCR5* were upregulated in *PRDM1* overexpressing human primary T cells, and effector markers including *GZMA* and *KLRD1* were upregulated in *PRDM1* overexpressing human primary T cells. Another study showed that *Prdm1* ^-/-^ Treg cells downregulated *Foxp3* and *Ctla4* (40), which is consistent with that we observed increased *FOXP3* expression level and increased chromatin accessibility of *CTLA4* in *PRDM1* overexpressing human primary T cells. Moreover, we found PRDM1 remodeled the chromatin accessibility in the region of several key T cell regulation TFs binding sites, such as JUNB, AP-1, BATF, which suggests that PRDM1 might act as a core TF that first altering the chromatin accessibility of key T cell regulatory TFs binding sites, then TFs bind to those accessible target genes and regulate the genes expression. These results consistent with recent work by Yoshikawa et al. which is PRDM1 regulating T cell chromatin accessibility in human engineered T cells (41). However, due to the limitation of cell numbers, we did not analyze the regulation network of PRDM1 in human primary CD4^+^ T cells and CD8^+^ T cells separately. Considering the different characteristics of CD4^+^ and CD8^+^ T cells, it is still necessary to investigate the PRDM1 regulation network in specific cell subsets.

*Prdm1*-mediated stability of *Foxp3* expression maintains Treg cell function (42, 43). Previous studies in mouse models showed that PRDM1 cooperated with IRF4 to control the differentiation and function of effector Treg cells (44). Further analysis demonstrated that PRDM1 maintained the CNS2 region of the *Foxp3* gene in a demethylated state and supported Treg cell identity and suppressive function (10). However, another study showed that PRDM1 limited the number of Treg cells (9). The regulatory mechanism of PRDM1 on FOXP3 is still controversial in mouse models. Here in our study, we found *FOXP3* was coexpressed with *PRDM1* in human T cells which is *FOXP3* was upregulated in *PRDM1* overexpressed T cells and downregulated in *PRDM1* knockdown T cell. We also found that PRDM1 binding to the upstream enhancer of *FOXP3* locus. Previous studies demonstrated that TFs can bring an enhancer into physical proximity with a promoter by the three-dimensional (3D) looping of DNA (45, 46). Recently, Kawakami et al. revealed that two independently activated enhancers, Foxp3-CNS0 and Foxp3-CNS3, cooperatively induce and maintain *Foxp3* expression for the establishment of self-tolerance in mouse models (47). The Foxp3-CNS0 enhancer in the mouse model is close to the position of the human *FOXP3* enhancer we found but does not overlap. Therefore, our results and related evidence in mouse models demonstrated that PRDM1 might directly activate *FOXP3* expression level by bring upstream enhancer into physical proximity with *FOXP3* promoter.

The necessary and sufficient role of PRDM1 in T cell tolerance induction and maintenance has been identified by our and previous studies. It might be helpful to upregulate *PRDM1* expression levels in T cells to treat immune diseases such as GVHD. However, no specific PRDM1 agonist has been reported. Pan-HDAC inhibitors have been reported to elevate *PRDM1* expression levels in follicular lymphoma cells (33), and we also found that another pan-HDAC inhibitor, PCI-24781, could elevate *PRDM1* expression levels in T cell acute lymphoblastic leukemia (T-ALL) cells. We also treated human primary T cells with PCI-24781 but found that it had little effect on upregulated *PRDM1* expression levels *in vitro*. Considering the master role of PRDM1 in the induction of T cell hyporesponsiveness, it is worth developing a specific agonist of PRDM1.

In summary, our study provides a global regulatory model of PRDM1 in human primary T cells. We present PRDM1 as a sufficient regulator in the induction of T cell hyporesponsiveness that alters chromatin accessibility, directly upregulates T cell inhibitory signals and downregulates T cell activation signals. In addition, our study provides new evidence for PRDM1 regulating *FOXP3* expression level in human T cells.

## Data Availability Statement

The datasets presented in this study can be found in online repositories. The names of the repository/repositories and accession number(s) can be found below: https://db.cngb.org/, accession ID: CNP0002725.

## Ethics Statement

The studies involving human participants were reviewed and approved by Ethics Committee of Peking University People’s Hospital. The patients/participants provided their written informed consent to participate in this study.

## Author Contributions

X-JH designed the study. X-JH and HG conceived the project and drafted the manuscript. HG and MW performed the experiments. All authors contributed to data interpretation and manuscript preparation.

## Funding

This work was partly supported by grants from the National Key Research and Development Program of China (2017YFA0104500), Innovative Research Groups of the National Natural Science Foundation of China (No. 81621001), Key Program of the National Natural Science Foundation of China (No.81530046, 81930004), Beijing Municipal Natural Science Foundation (Grant No. 7204319).

## Conflict of Interest

The authors declare that the research was conducted in the absence of any commercial or financial relationships that could be construed as a potential conflict of interest.

## Publisher’s Note

All claims expressed in this article are solely those of the authors and do not necessarily represent those of their affiliated organizations, or those of the publisher, the editors and the reviewers. Any product that may be evaluated in this article, or claim that may be made by its manufacturer, is not guaranteed or endorsed by the publisher.

## References

[1] Zhang P Lee JS Gartlan KH Schuster IS Comerford I Varelias A. Eomesodermin Promotes the Development of Type 1 Regulatory T (TR1) Cells. Sci Immunol (2017) 2(10):eaah7152. doi: 10.1126/sciimmunol.aah7152 28738016 PMC5714294

[2] Shapiro-Shelef M Lin KI McHeyzer-Williams LJ Liao J McHeyzer-Williams MG Calame K. Blimp-1 Is Required for the Formation of Immunoglobulin Secreting Plasma Cells and Pre-Plasma Memory B Cells. Immun (2003) 19(4):607–20. doi: 10.1016/s1074-7613(03)00267-x 14563324

[3] Shaffer AL Lin KI Kuo TC Yu X Hurt EM Rosenwald A. Blimp-1 Orchestrates Plasma Cell Differentiation by Extinguishing the Mature B Cell Gene Expression Program. Immun (2002) 17(1):51–62. doi: 10.1016/s1074-7613(02)00335-7 12150891

[4] Fu SH Yeh LT Chu CC Yen BL Sytwu HK. New Insights Into Blimp-1 in T Lymphocytes: A Divergent Regulator of Cell Destiny and Effector Function. J BioMed Sci (2017) 24(1):49. doi: 10.1186/s12929-017-0354-8 28732506 PMC5520377

[5] Bikoff EK Morgan MA Robertson EJ. An Expanding Job Description for Blimp-1/Prdm1. Curr Opin Genet Dev (2009) 19(4):379–85. doi: 10.1016/j.gde.2009.05.005 19592232

[6] Nutt SL Fairfax KA Kallies A. BLIMP1 Guides the Fate of Effector B and T Cells. Nat Rev Immunol (2007) 7(12):923–7. doi: 10.1038/nri2204 17965637

[7] Kallies A Hawkins ED Belz GT Metcalf D Hommel M Corcoran LM. Transcriptional Repressor Blimp-1 Is Essential for T Cell Homeostasis and Self-Tolerance. Nat Immunol (2006) 7(5):466–74. doi: 10.1038/ni1321 16565720

[8] Martins GA Cimmino L Shapiro-Shelef M Szabolcs M Herron A Magnusdottir E. Transcriptional Repressor Blimp-1 Regulates T Cell Homeostasis and Function. Nat Immunol (2006) 7(5):457–65. doi: 10.1038/ni1320 16565721

[9] Cretney E Xin A Shi W Minnich M Masson F Miasari M. The Transcription Factors Blimp-1 and IRF4 Jointly Control the Differentiation and Function of Effector Regulatory T Cells. Nat Immunol (2011) 12(4):304–11. doi: 10.1038/ni.2006 21378976

[10] Garg G Muschaweckh A Moreno H Vasanthakumar A Floess S Lepennetier G. Blimp1 Prevents Methylation of Foxp3 and Loss of Regulatory T Cell Identity at Sites of Inflammation. Cell Rep (2019) 26(7):1854–1868.e5. doi: 10.1016/j.celrep.2019.01.070 30759395 PMC6389594

[11] Buenrostro JD Giresi PG Zaba LC Chang HY Greenleaf WJ. Transposition of Native Chromatin for Fast and Sensitive Epigenomic Profiling of Open Chromatin, DNA-Binding Proteins and Nucleosome Position. Nat Methods (2013) 10(12):1213–8. doi: 10.1038/nmeth.2688 PMC3959825 24097267

[12] Buenrostro JD Wu B Chang HY Greenleaf WJ. ATAC-Seq: A Method for Assaying Chromatin Accessibility Genome-Wide. Curr Protoc Mol Biol (2015) 109:21:29 1–21 29 9. doi: 10.1002/0471142727.mb2129s109 25559105 PMC4374986

[13] Kaya-Okur HS Wu SJ Codomo CA Pledger ES Bryson TD Henikoff JG. CUT&Tag for Efficient Epigenomic Profiling of Small Samples and Single Cells. Nat Commun (2019) 10(1):1930. doi: 10.1038/s41467-019-09982-5 31036827 PMC6488672

[14] Kaya-Okur HS Janssens DH Henikoff JG Ahmad K Henikoff S. Efficient Low-Cost Chromatin Profiling With CUT&Tag. Nat Protoc (2020) 15(10):3264–83. doi: 10.1038/s41596-020-0373-x PMC8318778 32913232

[15] Filipovich AH Weisdorf D Pavletic S Socie G Wingard JR Lee SJ. National Institutes of Health Consensus Development Project on Criteria for Clinical Trials in Chronic Graft-Versus-Host Disease: I. Diagnosis and Staging Working Group Report. Biol Blood Marrow Transplant (2005) 11(12):945–56. doi: 10.1016/j.bbmt.2005.09.004 16338616

[16] Przepiorka D Weisdorf D Martin P Klingemann HG Beatty P Hows J. 1994 Consensus Conference on Acute GVHD Grading. Bone Marrow Transplant (1995) 15(6):825–8. 7581076

[17] Ma J Nie K Redmond D Liu Y Elemento O Knowles DM. EBV-miR-BHRF1-2 Targets PRDM1/Blimp1: Potential Role in EBV Lymphomagenesis. Leukemia (2016) 30(3):594–604. doi: 10.1038/leu.2015.285 26530011 PMC4777778

[18] Pertea M Kim D Pertea GM Leek JT Salzberg SL. Transcript-Level Expression Analysis of RNA-Seq Experiments With HISAT, StringTie and Ballgown. Nat Protoc (2016) 11(9):1650–67. doi: 10.1038/nprot.2016.095 PMC5032908 27560171

[19] Chen Z Arai E Khan O Zhang Z Ngiow SF He Y. *In Vivo* CD8(+) T Cell CRISPR Screening Reveals Control by Fli1 in Infection and Cancer. Cell (2021) 184(5):1262–1280.e22. doi: 10.1016/j.cell.2021.02.019 33636129 PMC8054351

[20] Baron U Floess S Wieczorek G Baumann K Grutzkau A Dong J. DNA Demethylation in the Human FOXP3 Locus Discriminates Regulatory T Cells From Activated FOXP3(+) Conventional T Cells. Eur J Immunol (2007) 37(9):2378–89. doi: 10.1002/eji.200737594 17694575

[21] Marrone G Maeso-Diaz R Garcia-Cardena G Abraldes JG Garcia-Pagan JC Bosch J. KLF2 Exerts Antifibrotic and Vasoprotective Effects in Cirrhotic Rat Livers: Behind the Molecular Mechanisms of Statins. Gut (2015) 64(9):1434–43. doi: 10.1136/gutjnl-2014-308338 25500203

[22] Li R Guo H Liu S Wang M Peng T Zhao X-Y. Three-Dimensional Genome Structure and Chromatin Accessibility Reorganization During *In Vivo* Induction of Human T Cell Tolerance. bioRxiv (2020). doi: 10.1101/2020.03.11.988253

[23] Pabbisetty SK Rabacal W Volanakis EJ Parekh VV Olivares-Villagomez D Cendron D. Peripheral Tolerance Can Be Modified by Altering KLF2-Regulated Treg Migration. Proc Natl Acad Sci USA (2016) 113(32):E4662–70. doi: 10.1073/pnas.1605849113 PMC4987800 27462110

[24] Gustafson CE Qi Q Hutter-Saunders J Gupta S Jadhav R Newell E. Immune Checkpoint Function of CD85j in CD8 T Cell Differentiation and Aging. Front Immunol (2017) 8:692. doi: 10.3389/fimmu.2017.00692 28659925 PMC5469909

[25] Lebbink RJ Meyaard L. Non-MHC Ligands for Inhibitory Immune Receptors: Novel Insights and Implications for Immune Regulation. Mol Immunol (2007) 44(9):2153–64. doi: 10.1016/j.molimm.2006.11.014 17188357

[26] Blackburn SD Shin H Haining WN Zou T Workman CJ Polley A. Coregulation of CD8+ T Cell Exhaustion by Multiple Inhibitory Receptors During Chronic Viral Infection. Nat Immunol (2009) 10(1):29–37. doi: 10.1038/ni.1679 19043418 PMC2605166

[27] Andre P Denis C Soulas C Bourbon-Caillet C Lopez J Arnoux T. Anti-NKG2A mAb Is a Checkpoint Inhibitor That Promotes Anti-Tumor Immunity by Unleashing Both T and NK Cells. Cell (2018) 175(7):1731–1743.e13. doi: 10.1016/j.cell.2018.10.014 30503213 PMC6292840

[28] Kamiya T Seow SV Wong D Robinson M Campana D. Blocking Expression of Inhibitory Receptor NKG2A Overcomes Tumor Resistance to NK Cells. J Clin Invest (2019) 129(5):2094–106. doi: 10.1172/JCI123955 PMC6486333 30860984

[29] Patterson SJ Pesenacker AM Wang AY Gillies J Mojibian M Morishita K. T Regulatory Cell Chemokine Production Mediates Pathogenic T Cell Attraction and Suppression. J Clin Invest (2016) 126(3):1039–51. doi: 10.1172/JCI83987 PMC4767359 26854929

[30] Martins GA Cimmino L Liao J Magnusdottir E Calame K. Blimp-1 Directly Represses Il2 and the Il2 Activator Fos, Attenuating T Cell Proliferation and Survival. J Exp Med (2008) 205(9):1959–65. doi: 10.1084/jem.20080526 PMC2526191 18725523

[31] Schraml BU Hildner K Ise W Lee WL Smith WA Solomon B. The AP-1 Transcription Factor Batf Controls T(H)17 Differentiation. Nat (2009) 460(7253):405–9. doi: 10.1038/nature08114 PMC2716014 19578362

[32] Ise W Kohyama M Schraml BU Zhang T Schwer B Basu U. The Transcription Factor BATF Controls the Global Regulators of Class-Switch Recombination in Both B Cells and T Cells. Nat Immunol (2011) 12(6):536–43. doi: 10.1038/ni.2037 PMC3117275 21572431

[33] Desmots F Roussel M Pangault C Llamas-Gutierrez F Pastoret C Guiheneuf E. Pan-HDAC Inhibitors Restore PRDM1 Response to IL21 in CREBBP-Mutated Follicular Lymphoma. Clin Cancer Res (2019) 25(2):735–46. doi: 10.1158/1078-0432.CCR-18-1153 30348636

[34] Pabbisetty SK Rabacal W Maseda D Cendron D Collins PL Hoek KL. KLF2 Is a Rate-Limiting Transcription Factor That can be Targeted to Enhance Regulatory T-Cell Production. Proc Natl Acad Sci USA (2014) 111(26):9579–84. doi: 10.1073/pnas.1323493111 PMC4084438 24979767

[35] Wang AYL Loh CYY Chen SJ Kao HK Lin CH Chuang SH. Blimp-1 Prolongs Allograft Survival Without Regimen *via* Influencing T Cell Development in Favor of Regulatory T Cells While Suppressing Th1. Mol Immunol (2018) 99:53–65. doi: 10.1016/j.molimm.2018.04.004 29698799

[36] Cimmino L Martins GA Liao J Magnusdottir E Grunig G Perez RK. Blimp-1 Attenuates Th1 Differentiation by Repression of Ifng, Tbx21, and Bcl6 Gene Expression. J Immunol (2008) 181(4):2338–47. doi: 10.4049/jimmunol.181.4.2338 18684923

[37] Johnston RJ Poholek AC DiToro D Yusuf I Eto D Barnett B. Bcl6 and Blimp-1 are Reciprocal and Antagonistic Regulators of T Follicular Helper Cell Differentiation. Sci (2009) 325(5943):1006–10. doi: 10.1126/science.1175870 PMC2766560 19608860

[38] Gong D Malek TR. Cytokine-Dependent Blimp-1 Expression in Activated T Cells Inhibits IL-2 Production. J Immunol (2007) 178(1):242–52. doi: 10.4049/jimmunol.178.1.242 17182561

[39] Xin A Masson F Liao Y Preston S Guan T Gloury R. A Molecular Threshold for Effector CD8(+) T Cell Differentiation Controlled by Transcription Factors Blimp-1 and T-Bet. Nat Immunol (2016) 17(4):422–32. doi: 10.1038/ni.3410 PMC5779087 26950239

[40] Dixon ML Luo L Ghosh S Grimes JM Leavenworth JD Leavenworth JW. Remodeling of the Tumor Microenvironment *via* Disrupting Blimp1(+) Effector Treg Activity Augments Response to Anti-PD-1 Blockade. Mol Cancer (2021) 20(1):150. doi: 10.1186/s12943-021-01450-3 34798898 PMC8605582

[41] Yoshikawa T Wu Z Inoue S Kasuya H Matsushita H Takahashi Y. Genetic Ablation of PRDM1 in Antitumor T Cells Enhances Therapeutic Efficacy of Adoptive Immunotherapy. Blood (2022) 139(14):2156–72. doi: 10.1182/blood.2021012714 34861037

[42] Bailey-Bucktrout SL Martinez-Llordella M Zhou X Anthony B Rosenthal W Luche H. Self-Antigen-Driven Activation Induces Instability of Regulatory T Cells During an Inflammatory Autoimmune Response. Immun (2013) 39(5):949–62. doi: 10.1016/j.immuni.2013.10.016 PMC3912996 24238343

[43] Ogawa C Bankoti R Nguyen T Hassanzadeh-Kiabi N Nadeau S Porritt RA. Blimp-1 Functions as a Molecular Switch to Prevent Inflammatory Activity in Foxp3(+)RORgammat(+) Regulatory T Cells. Cell Rep (2018) 25(1):19–28.e5. doi: 10.1016/j.celrep.2018.09.016 30282028 PMC6237548

[44] Ohkura N Sakaguchi S. Maturation of Effector Regulatory T Cells. Nat Immunol (2011) 12(4):283–4. doi: 10.1038/ni0411-283 21423222

[45] Furlong EEM Levine M. Developmental Enhancers and Chromosome Topology. Sci (2018) 361(6409):1341–5. doi: 10.1126/science.aau0320 PMC6986801 30262496

[46] Kim S Shendure J. Mechanisms of Interplay Between Transcription Factors and the 3D Genome. Mol Cell (2019) 76(2):306–19. doi: 10.1016/j.molcel.2019.08.010 31521504

[47] Kawakami R Kitagawa Y Chen KY Arai M Ohara D Nakamura Y. Distinct Foxp3 Enhancer Elements Coordinate Development, Maintenance, and Function of Regulatory T Cells. Immunity (2021) 54(5):947–961.e8. doi: 10.1016/j.immuni.2021.04.005 33930308

