# Supplementary figures and images for: PRDM1 Drives Human Primary T Cell Hyporesponsiveness by Altering the T Cell Transcriptome and Epigenome
Source: Front Immunol. 2022 Apr 28;13:879501. doi: 10.3389/fimmu.2022.879501 (PMC9097451; doi:10.3389/fimmu.2022.879501)

Fig.S1

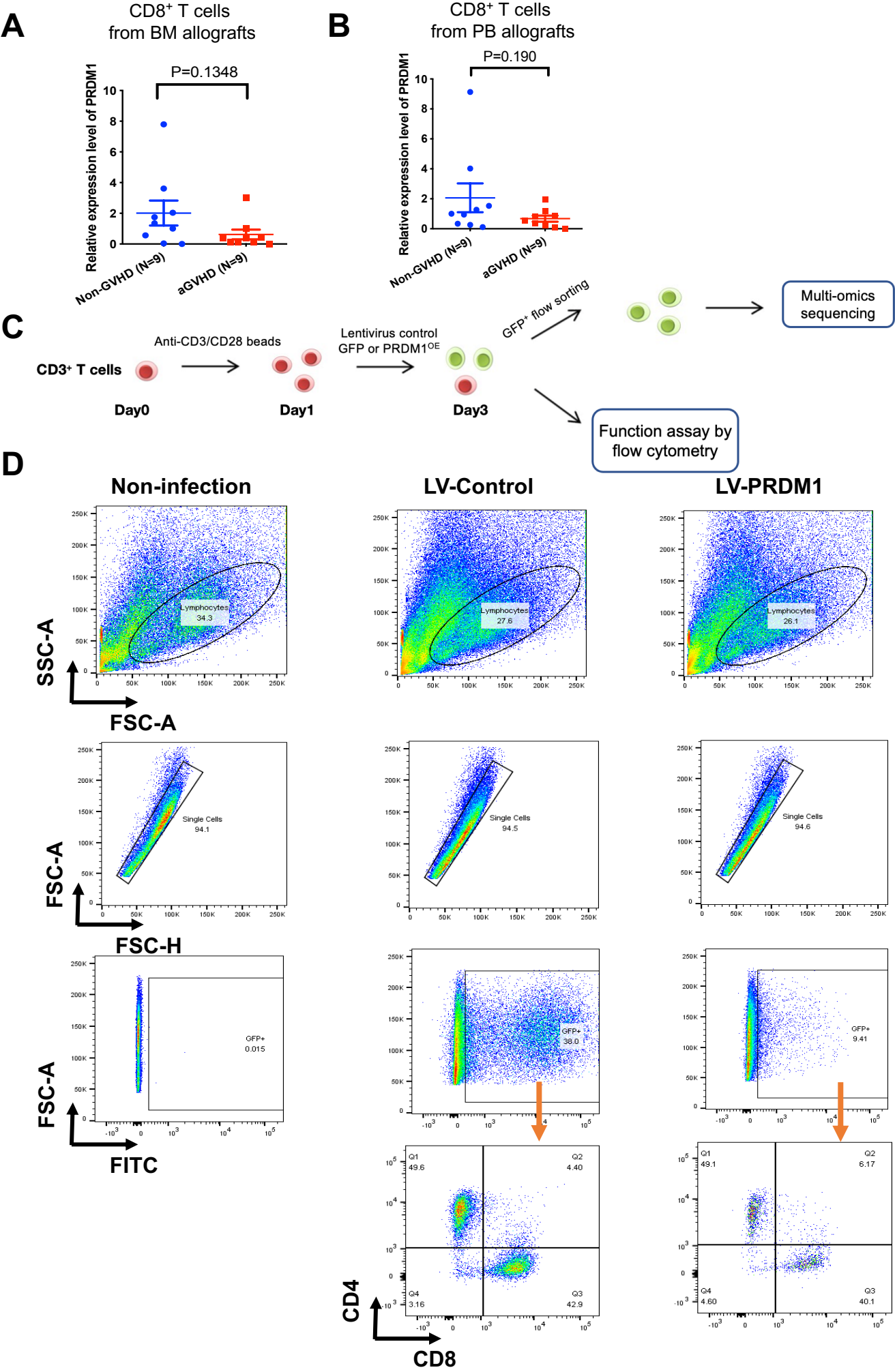

Fig.S2

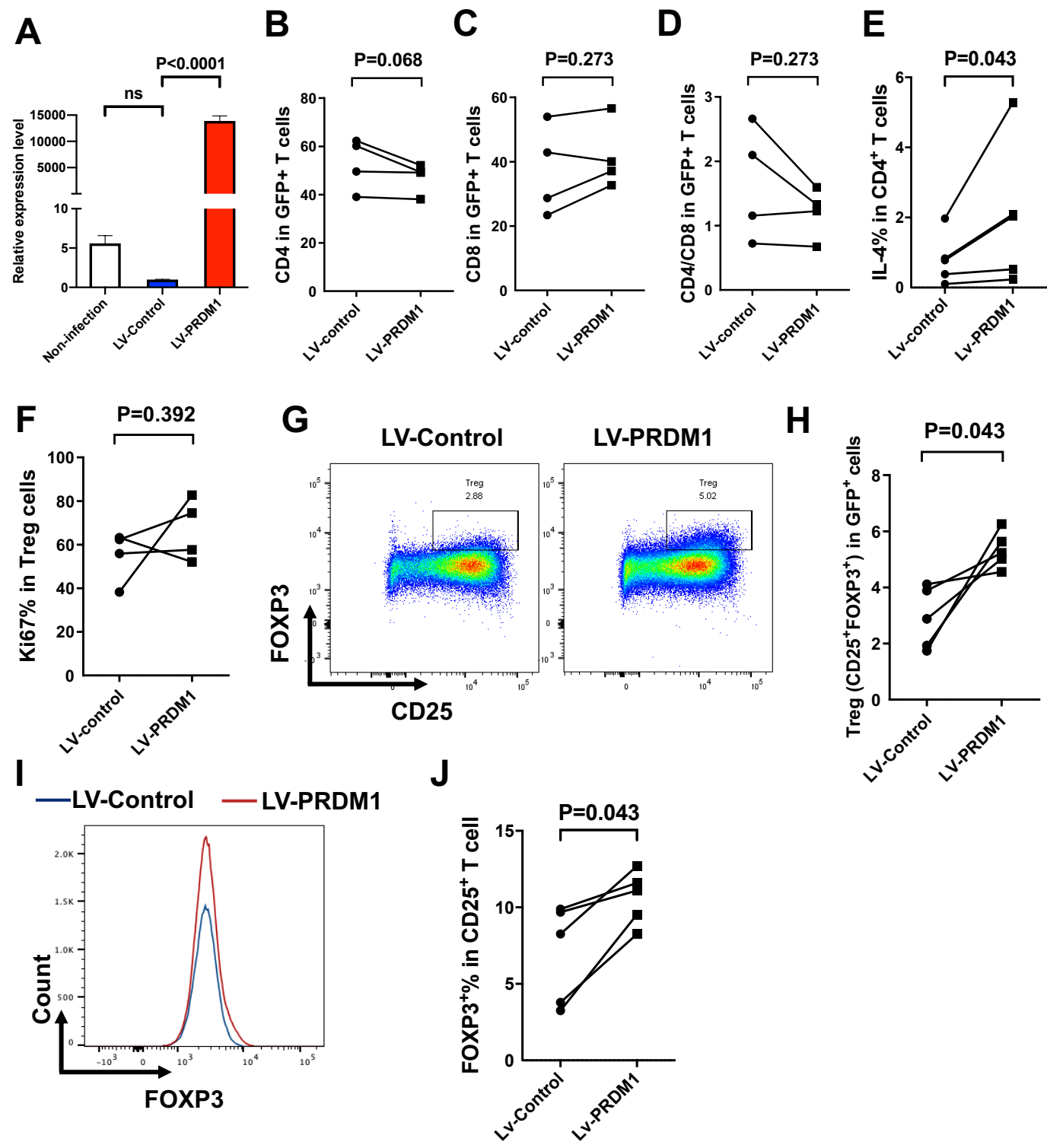

Fig.S3

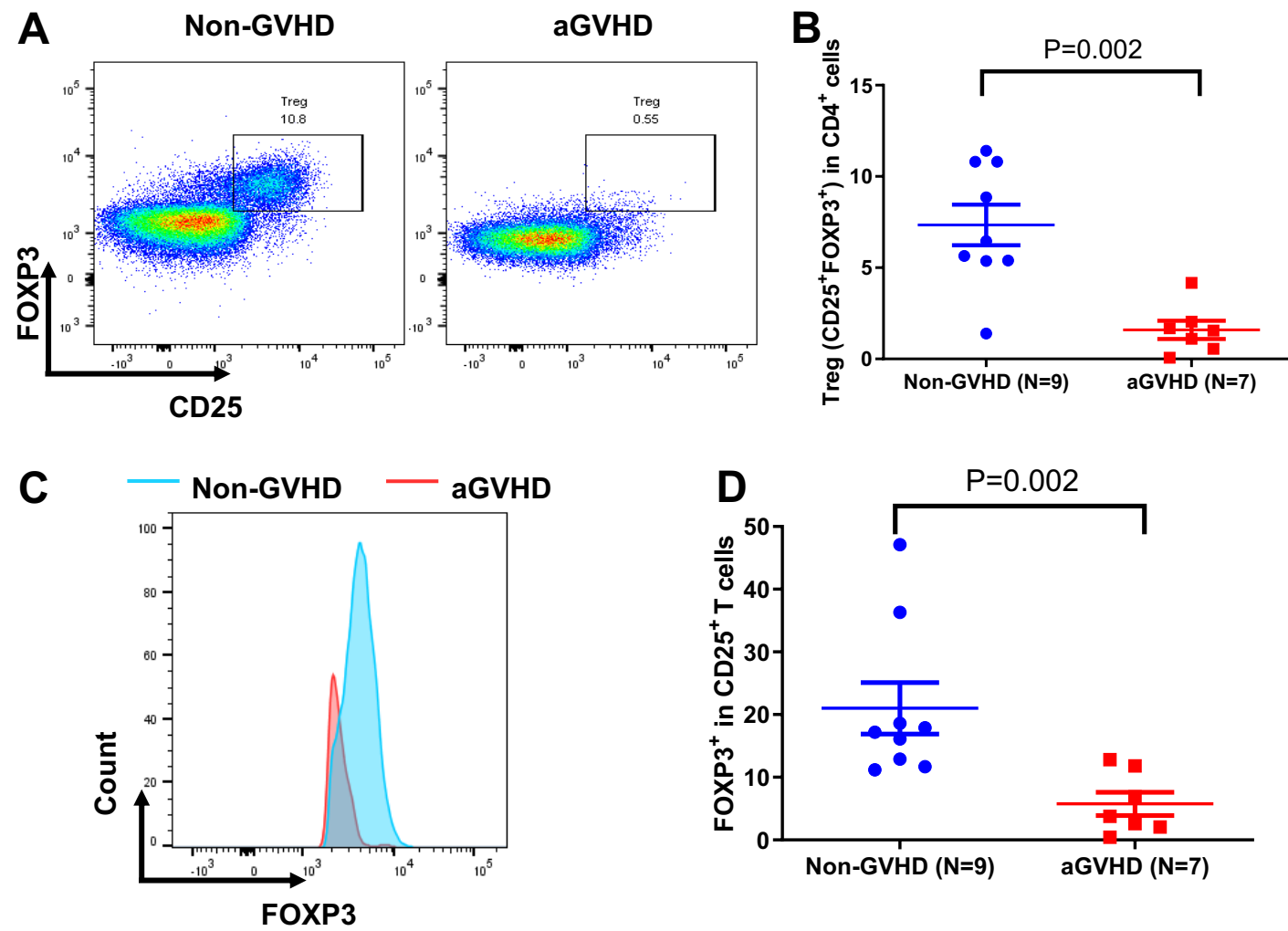

Fig.S4

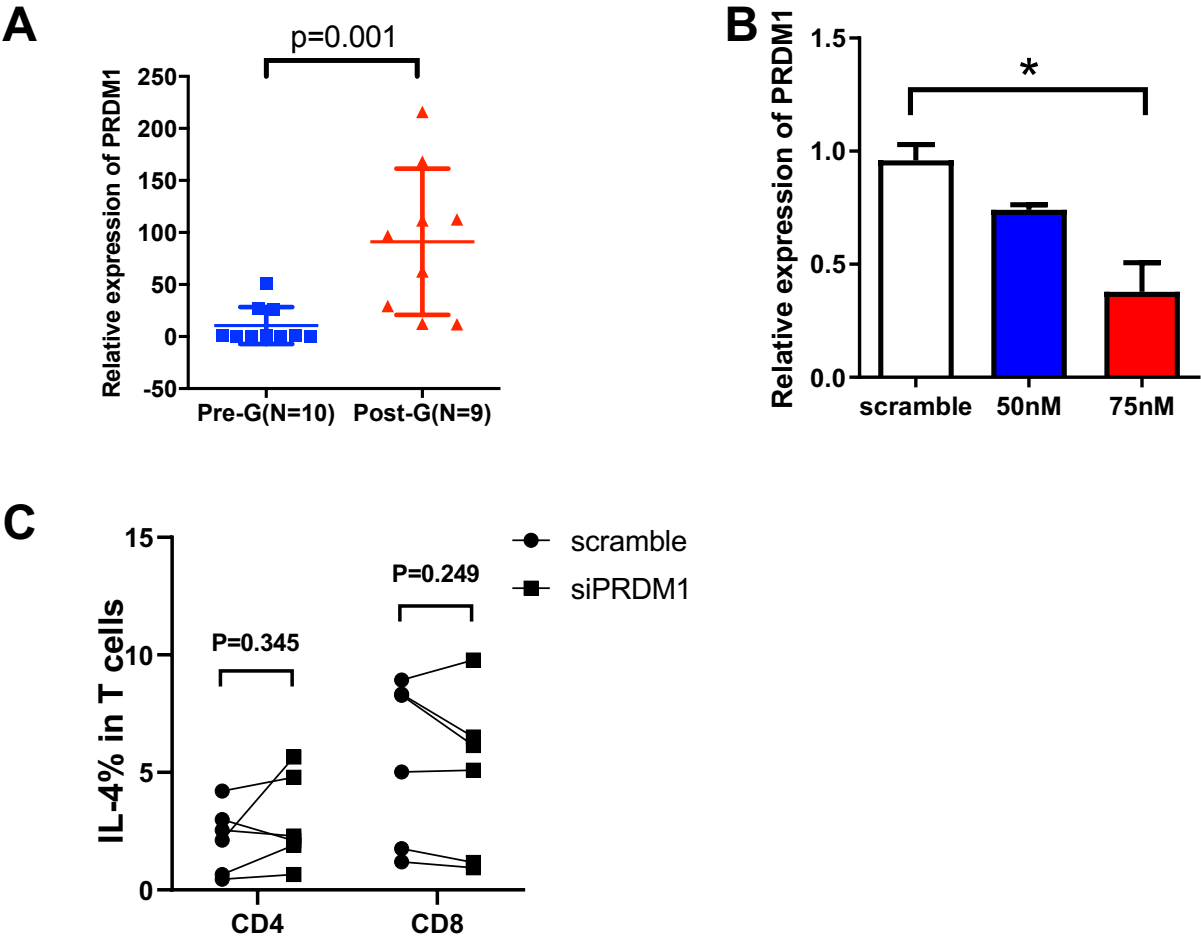

Fig.S5

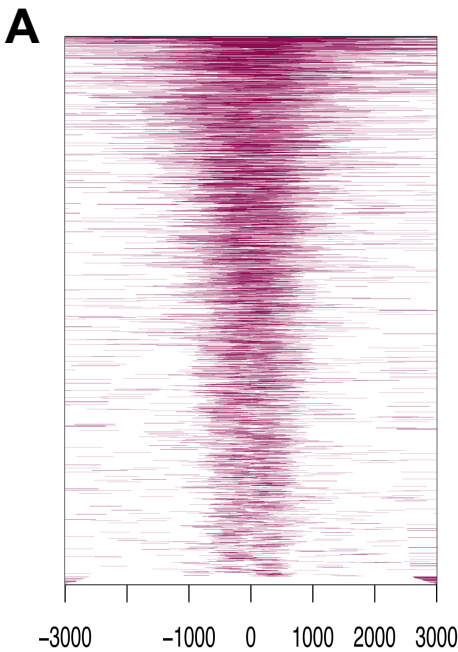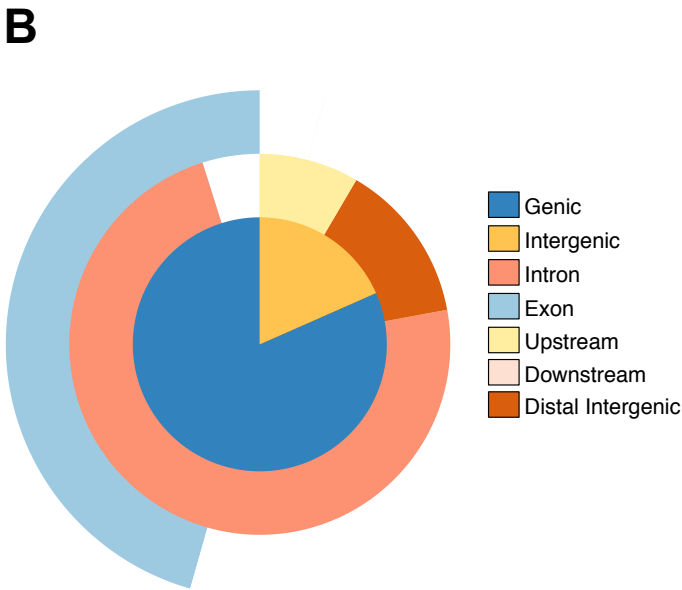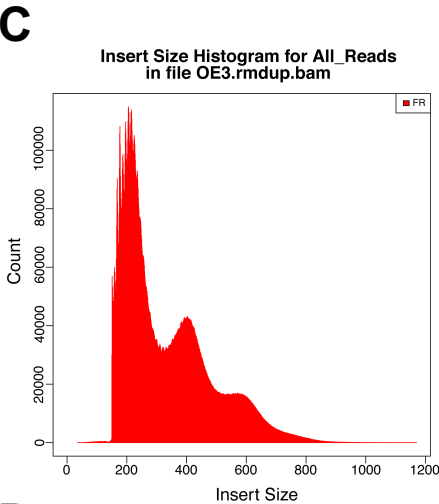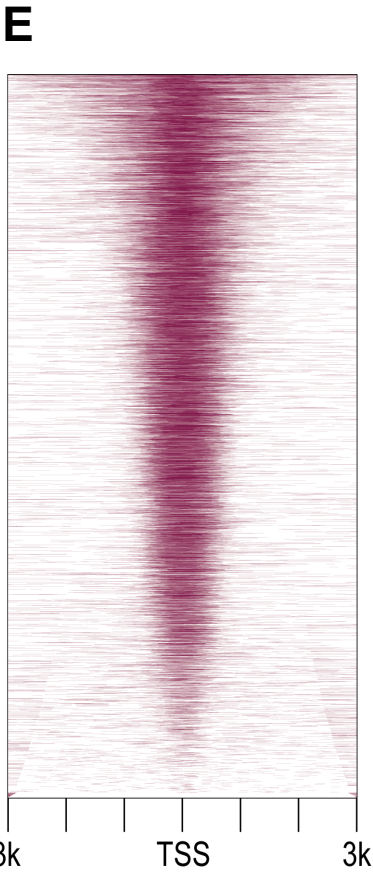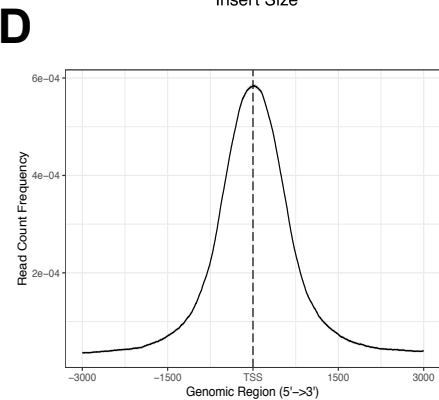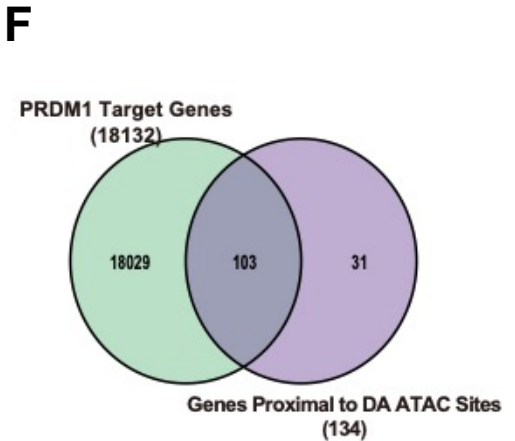

Fig.S6

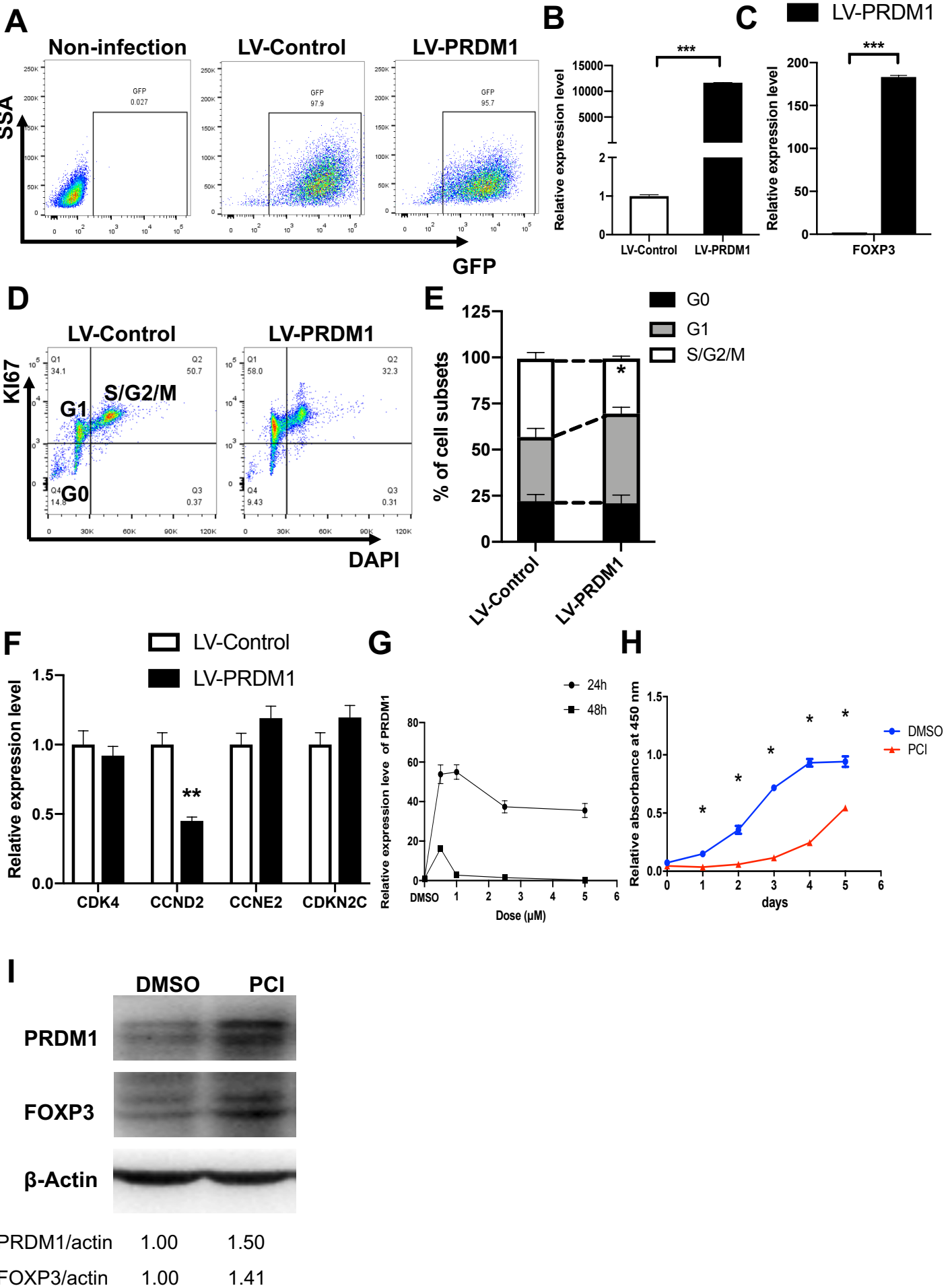

Fig.S7

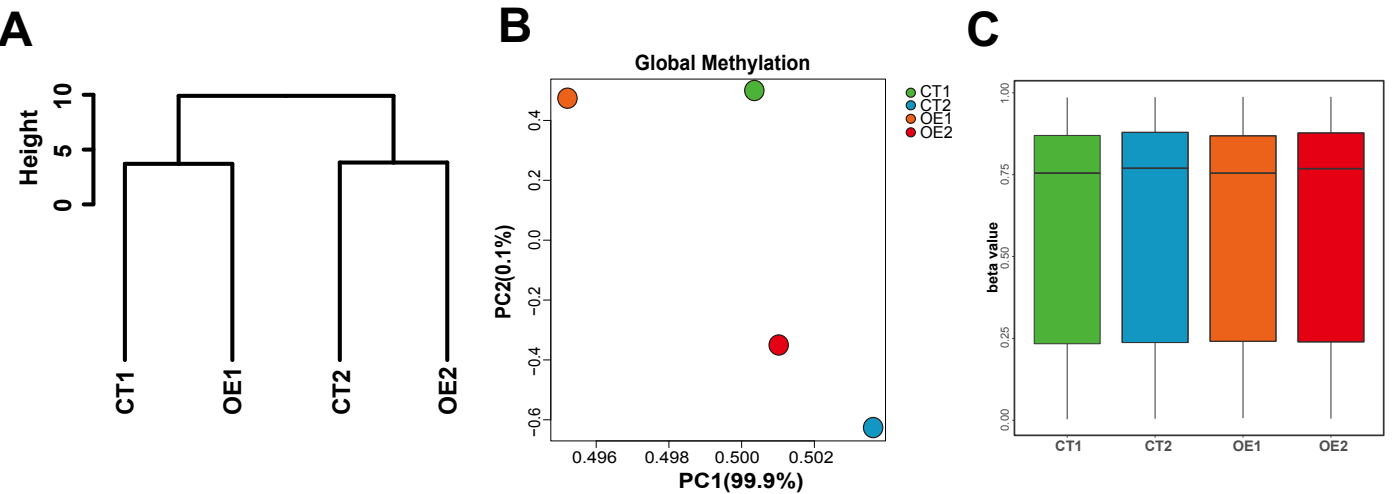

**D**

Position of selected amplicons on *FOXP3* gene region

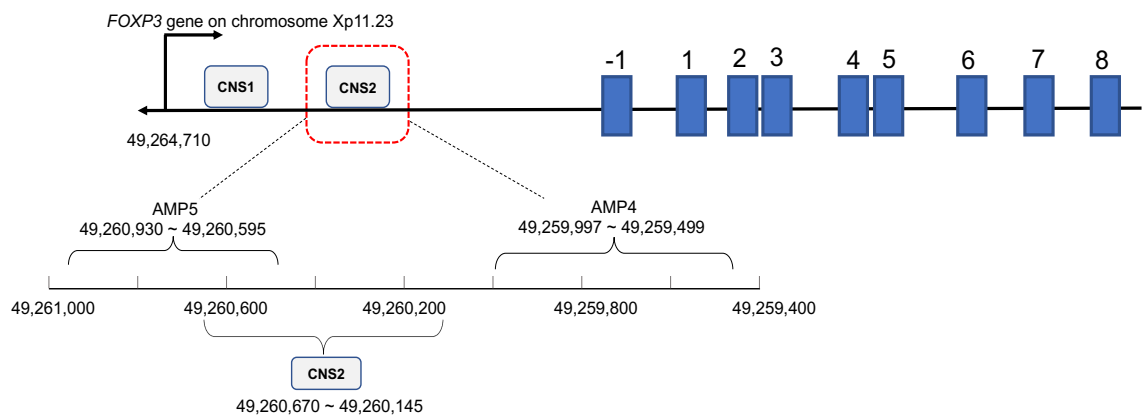

Supplement: Supplementary file 1 [file DataSheet_1.pdf]
