# Supplementary material for: PRDM1 Drives Human Primary T Cell Hyporesponsiveness by Altering the T Cell Transcriptome and Epigenome
Source: Front Immunol. 2022 Apr 28;13:879501. doi: 10.3389/fimmu.2022.879501 (PMC9097451; doi:10.3389/fimmu.2022.879501)
Supplement: Supplementary file 2 [file Table_1.docx]

**Supplementary** **Materials**

**Supplementary Figure**

**Fig. S1. Overexpressed *PRDM1* in human primary T cells.**

**(A-B)** Quantitative real-time PCR showed the expression level of *PRDM1* in CD8^+^ T cells from bone marrow allografts **(A)** or from peripheral allografts **(B)** and the aGVHD occurrence in related patients, respectively. Mann–Whitney U test was used. **(C)** Illustration of overexpressing experiment design. **(D)** Gating strategy for flow cytometry analysis.

**Fig. S2**. **Phenotype of *PRDM1* overexpression in primary T cells.**

**(A)** Quantitative real-time PCR was used to detect *PRDM1* expression level in *PRDM1*-overexpressing T cells or control T cells (n = 3). Student’s t test was used. **(B-D)** The ratio of CD4^+^ T cells **(B)**, CD8^+^ T cells **(C)** and CD4/CD8 **(D)** in GFP^+^ T cells. **(E)** Percentage of IL-4 secretion level of PRDM1 overexpressing CD8^+^ T cells compared with control T cells (n = 5). **(F)** Flow cytometry results showed the percentage of Ki67 level on *PRDM1* overexpressing Treg cells (n = 4). **(G-H)** Representative flow cytometry results **(G)** and percentages **(H)** showed the Treg cells in CD4^+^ conventional T cells (CD4^+^CD25^-^) infected with *PRDM1*-overexpressing lentivirus or GFP^+^ control (n = 5). **(I-J)** FOXP3 protein level in *PRDM1*-overexpressing GFP^+^CD4^+^CD25^+^ T cells compared with control T cells (n = 5). Wilcoxon rank sum test was performed to assess the significance. *P < 0.05.

**Fig. S3**. **Treg population and FOXP3 protein level in patients with or without aGVHD.**

**(A-B)** Representative flow cytometry results **(A)** and percentages **(B)** showed the Treg cells in CD4^+^ T cells from patients with or without aGVHD. **(C-D)** Representative flow cytometry results **(C)** and percentages **(D)** showed the FOXP3 protein level in CD4^+^CD25^+^ T cells from patients with or without aGVHD. Mann–Whitney U test was used.

**Fig. S4**. **Phenotype of *PRDM1* knockdown in primary T cells.**

**(A)** Quantitative real-time PCR was used to detect *PRDM1* expression level in G-CSF mobilized T cells compared with steady state T cells. Mann–Whitney U test was used. **(B)** Quantitative real-time PCR was used to detect *PRDM1* expression level in *PRDM1* knocked down T cells by different concentration of siRNA (n = 3). Student’s t test was used. **(C)** Percentage of IL-4 secretion in *PRDM1* knocked down CD4^+^ T cells and CD8^+^ T cells compared with control T cells (n = 6). Wilcoxon rank sum test was performed to assess the significance. *P < 0.05.

**Fig. S5**. **Quality control of CUT&Tag and ATAC-seq data from *PRDM1* overexpressed T cells.**

**(A)** Peaks distribution around transcription start sites (TSS) of CUT&Tag data. **(B)** Peaks annotation in whole genome of CUT&Tag data. **(C)** Insert size histogram for all reads in *PRDM1*-overexpressing T cells of ATAC-seq data. **(D-E)** Peaks distribution around transcription start sites (TSS) of ATAC-seq data. **(F)** The overlap between PRDM1 target genes and different accessibility proximal genes in *PRDM1*-overexpressing T cells compared with control T cells.

**Fig. S6**. **PRDM1 inhibited Jurkat cell proliferation and elevated FOXP3 expression.**

**(A)** Representative flow cytometry results of lentivirus infected Jurkat T cells. **(B-C)** Quantitative real-time PCR was used to detect *PRDM1* **(B)** and *FOXP3* **(C)** expression level in *PRDM1*-overexpressing Jurkat T cells or control groups. **(D-E)** Representative flow cytometry results **(D)** and percentages **(E)** of cell cycle in *PRDM1*-overexpressing Jurkat T cells compared with control groups. **(F)** Quantitative real-time PCR was used to detect cell cycle regulation genes expression level in *PRDM1* overexpressing Jurkat T cells. Student’s t test was used. **(G)** Quantitative real-time PCR was used to detect *PRDM1* expression level in PCI-24781 treated Jurkat T cells in different concentration and time. **(H)** CCK8 analysis showed the proliferation of PCI-24781 treatment Jurkat T cells. **(I)** PRDM1 protein level in PCI-24781 treatment Jurkat cells compared with DMSO treatment Jurkat T cells. Student’s t test was used. *P < 0.05, **P < 0.01, ***P < 0.001

**Fig. S7. PRDM1 did not change the methylation level of the genome of human primary T cells.**

**(A)** Unsupervised hierarchical clustering of the beta values for *PRDM1*-overexpressing T cells and control T cells. **(B)** PCA plot of the beta values for *PRDM1*-overexpressing T cells and control T cells. **(C)** Boxplot of the beta values for *PRDM1*-overexpressing T cells and control T cells. **(D)** Schematic of the detected position on *FOXP3* gene region by pyrosequencing.

**Table S1.** Characteristics of donors and patients with aGVHD and without aGVHD (related to Fig. 1A-B, Fig. S1A-B).

| **Characteristics** | **aGVHD group (N=9)** | **Non-GVHD group (N=9)** | ***P-*Value*** |
| --- | --- | --- | --- |
| Donor gender |  |  | 0.62 |
| Male | 7 | 5 |  |
| Female | 2 | 4 |  |
| Donor age, median(range) | 35.5(16-54) | 39(16-63) | 0.436 |
| Patient gender |  |  | 0.62 |
| Male | 5 | 7 |  |
| Female | 4 | 2 |  |
| Patient age, median(range) | 25(7-62) | 29(6-56) | 0.136 |
| Underlying disease |  |  | 0.70 |
| AML | 2 | 1 |  |
| ALL | 6 | 6 |  |
| Others | 1 | 2 |  |
| Source of stem cell |  |  | 1.00 |
| BM and PB | 9 | 9 |  |
| Donor match |  |  | 1.00 |
| HLA-matched sibling donor | 1 | 1 |  |
| Haploidentical related donor | 8 | 8 |  |
| Transplanted total nucleated cell dose (×10^8^/ kg) | 11.27(8.04-12.34) | 9.68(7.39-13.88) | 0.34 |
| Transplanted BM nucleated cell dose (×10^8^/ kg) | 4.47(3.00-6.22) | 2.36(2.00-3.49) | 0.001 |
| Transplanted PB nucleated cell dose (×10^8^/ kg) | 6.61(2.31-9.28) | 6.37(5.00-10.39) | 1.00 |
| Transplanted CD34^+^ cell dose (×10^6^/ kg) | 2.45(1.47-5.88) | 3.15(1.04-6.29) | 0.796 |
| Transplanted CD3^+^ cell dose (×10^8^/ kg) | 2.35 (0.14-6.08) | 3.40(0.37-6.52) | 0.55 |
| Transplanted CD4^+^ cell dose (×10^8^/ kg) | 2.12(0.11-3.84) | 2.00(0.34-7.36) | 0.73 |
| Transplanted CD8^+^ cell dose (×10^8^/ kg) | 0.30(0.09-0.75) | 0.26(0.12-0.52) | 0.67 |
| Transplanted CD14^+^ cell dose (×10^8^/ kg) | 1.57(0.11-2.51) | 1.75(0.29-3.81) | 0.49 |
| Conditioning |  |  | 1.00 |
| BU/CY | 1 | 1 |  |
| BU/CY+ATG | 8 | 8 |  |

*****Continuous variables were compared using the Mann-Whitney U test; categorical variables were compared using Fisher's exact test. P < 0.05 was considered significant.

Abbreviations: BM, bone marrow; PB, peripheral blood; AML, acute myelogenous leukemia; ALL, acute lymphocytic leukemia; ATG, antithymocyte globulin; aGVHD, acute graft-versus-host disease.

**Table S2.** Characteristics of allo-HSCT patients with aGVHD and without aGVHD (related to Fig.1C)

| **Characteristics** | **aGVHD (N=7)** | **Non-aGVHD(N=7)** | ***P-*Value*** |
| --- | --- | --- | --- |
| Days post-HSCT | 35(13-66) | 28(18-86) | 0.70 |
| Blood cell count |  |  |  |
| Median WBC (×10^9^/L) (range) | 4.70(1.98-19.27) | 5.02(1.60-11.10) | 0.62 |
| Median ANC (×10^9^/L) (range) | 4.11(1.68-17.40) | 3.71(1.13-8.59) | 0.32 |
| Median Hb (g/L) (range) | 88(57-96) | 93(64-109) | 0.26 |
| Median PLT (×10^9^/L) (range) | 44(26-123) | 71(19-206) | 0.54 |
| Age at HSCT (years, median, range) | 33(12-65) | 30(11-63) | 0.54 |
| Gender (male/female) | 4/3 | 5/2 | 0.89 |
| Underlying disease |  |  | 1.00 |
| AML | 3 | 4 |  |
| ALL | 4 | 3 |  |
| MDS | 0 | 0 |  |
| Status at HSCT |  |  | 1.00 |
| Standard-risk | 4 | 3 |  |
| High-risk | 3 | 4 |  |
| Source of stem cell |  |  | 1.00 |
| PB | 7 | 7 |  |
| Transplanted total nucleated cell dose (×10^8^/ kg, median, range) | 8.69 (7.7-13.01) | 10.64(7.01-13.97) | 0.90 |
| Transplanted CD34^+^ cell dose (×10^6^/ kg, median, range) | 2.98(1.52-6.72) | 3.60(1.66-4.81) | 0.62 |
| Donor match |  |  | 1.00 |
| HLA-identical sibling donor | 1 | 2 |  |
| HLA-partially matched related donor | 6 | 5 |  |
| Donor gender |  |  | 1.00 |
| Female | 2 | 1 |  |
| male | 5 | 6 |  |
| Donor-recipient pair |  |  | 1.00 |
| Female to male | 1 | 1 |  |
| Others | 6 | 6 |  |
| Blood type matching |  |  | 0.29 |
| Match | 5 | 2 |  |
| Mismatch | 2 | 5 |  |
| Pre-HSCT cycles of chemotherapy | 4 (2-5) | 5(3-8) | 0.36 |
| Conditioning |  |  | 1.00 |
| BU/CY | 2 | 2 |  |
| BU/CY+ATG | 5 | 5 |  |
| History of CMV reactivation | 2 | 4 | 1.00 |
| Onset of CMV reactivation (days, median, range) | 0(0-49) | 0(0-34) | 0.71 |

*****Continuous variables were compared using the Mann-Whitney U test; categorical variables were compared using Fisher's exact test. P < 0.05 was considered significant.

**Abbreviations:** allo-HSCT indicates allogeneic hematopoietic stem cell transplantation; aGVHD, acute graft-versus-host disease; PB, peripheral blood; WBC, white blood cell; ANC, absolute neutrophil cell; Hb, hemoglobin; PLT, platelet; AML, acute myelogenous leukemia; ALL, acute lymphocytic leukemia; HLA, human leukocyte antigen; ATG, antithymocyte globulin; CMV, cytomegalovirus.

**Table S3.** Characteristics of allo-HSCT patients with aGVHD and without aGVHD (related to Fig.S3)

| **Characteristics** | **aGVHD (N=7)** | **Non-aGVHD(N=9)** | ***P-*Value*** |
| --- | --- | --- | --- |
| Days post-HSCT | 47(18-91) | 21(13-64) | 0.09 |
| Blood cell count |  |  |  |
| Median WBC (×10^9^/L) (range) | 2.49(0.11-8.36) | 3.84(1.27-18.45) | 025 |
| Median ANC (×10^9^/L) (range) | 1.3(0.01-6.29) | 2.38(0.72-16.67) | 0.25 |
| Median Hb (g/L) (range) | 86(60-108) | 80(61-91) | 0.47 |
| Median PLT (×10^9^/L) (range) | 42(14-148) | 44(5-169) | 0.84 |
| Age at HSCT (years, median, range) | 49(18-57) | 41(19-61) | 0.54 |
| Gender (male/female) | 5/2 | 7/2 | 1.00 |
| Underlying disease |  |  | 0.93 |
| AML | 4 | 6 |  |
| ALL | 1 | 1 |  |
| MDS | 2 | 2 |  |
| Status at HSCT |  |  | 1.00 |
| Standard-risk | 4 | 6 |  |
| High-risk | 3 | 3 |  |
| Source of stem cell |  |  | 1.00 |
| PB | 7 | 9 |  |
| Transplanted total nucleated cell dose (×10^8^/ kg, median, range) | 10.87 (4.4-16.27) | 8.84(3.89-15.66) | 0.35 |
| Transplanted CD34^+^ cell dose (×10^6^/ kg, median, range) | 3.72(0.64-20.03) | 1.00(1.00-6.10) | 0.47 |
| Donor match |  |  | 1.00 |
| HLA-identical sibling donor | 2 | 3 |  |
| HLA-partially matched related donor | 5 | 6 |  |
| Donor gender |  |  | 0.62 |
| Female | 3 | 3 |  |
| male | 4 | 6 |  |
| Donor-recipient pair |  |  | 0.55 |
| Female to male | 2 | 1 |  |
| Others | 5 | 8 |  |
| Blood type matching |  |  | 1.00 |
| Match | 4 | 6 |  |
| Mismatch | 3 | 3 |  |
| Pre-HSCT cycles of chemotherapy | 2(0-4) | 2(0-4) | 0.84 |
| Conditioning |  |  | 0.63 |
| MAC | 5 | 5 |  |
| RIC | 2 | 4 |  |
| History of CMV reactivation | 4 | 1 | 0.11 |
| Onset of CMV reactivation (days, median, range) | 24(0-67) | 0(0-40) | 0.17 |

*****Continuous variables were compared using the Mann-Whitney U test; categorical variables were compared using Fisher's exact test. P < 0.05 was considered significant.

**Abbreviations:** allo-HSCT indicates allogeneic hematopoietic stem cell transplantation; aGVHD, acute graft-versus-host disease; PB, peripheral blood; WBC, white blood cell; ANC, absolute neutrophil cell; Hb, hemoglobin; PLT, platelet; AML, acute myelogenous leukemia; ALL, acute lymphocytic leukemia; MDS, Myelodysplastic syndromes; HLA, human leukocyte antigen; MAC, reduced-intensity conditioning; RIC, myeloablative conditioning; CMV, cytomegalovirus.

**Table S4**. Antibody information.

| Manufacturer | Name | Format | Clone | Cat. | Species |
| --- | --- | --- | --- | --- | --- |
| BD Pharmingen  BD Horizon  BD Horizon  BD Pharmingen  BD Pharmingen  BD Horizon  BD Pharmingen  CST  CST  CST  CST | CD4  CD8  CD8  Ki-67  IL-4  CD25  FOXP3  PRDM1  FOXP3  β-Actin  IgG | Percp-Cy5.5  APC-R700  V500  PE  APC  APC  PE | RPA-T4  RPA-T8  L50-823  B56  8D4-8  2A3  259D/C7  C14A4  D25D4  79D7  polyclone | 560650  565165  560074  556027  560671  340938  560046  9115  5298  100730  2729 | Human  Human  Human  Human  Human  Human  Human  Human  Human  Human  Human |

**Table S5**. Primer sequences.

| Primer name | Forward primer (5' to 3') | Reverse primer (5' to 3') |
| --- | --- | --- |
| *PRDM1* | TACATACCAAAGGGCACACG | TGAAGCTCCCCTCTGGAATA |
| *FOXP3* | GGCACAATGTCTCCTCCAGAGA | CAGATGAAGCCTTGGTCAGTGC |
| *KLF2* | CCAAGAGTTCGCATCTGAAGGC | CCGTGTGCTTTCGGTAGTGGC |
| *KIR3DL1* | GCAGGGAACAGAACAGCC | CCGTGTACAAGATGGTATCTGTA |
| *LILRB1* | CTCCCTATGAGTGGTCTCTACC | CTGTTGTAGCCAGCATCAGAGC |
| *KLRB1* | GTTCCACCAAAGAATCCAGCCTG | AAGAGCCGTTTATCCACTTCCAG |
| *2B4* | GTATTTGATAAAGTTGAGAAACCCCG | GCAGGTATATGTGTGAGTGCCATTA |
| *FOXD1* | TGAGCACTGAGATGTCCGATG | CACCACGTCGATGTCTGTTTC |
| *TOX* | CGCTACCTTTGGCGAAGTCTCT | CTGGCTCTGTATGCTGCGAGTT |
| *IFNGR2* | CTCCATTCTGCCTGGGTGACAA | CGTGGAGGTATCAGCGATGTCA |
| *ROMO1* | ATGGGCTTCGTGATGGGTTGCG | GCCACTCTGCATCATGGTTTTCC |
| *IL2* | AGAACTCAAACCTCTGGAGGAAG | GCTGTCTCATCAGCATATTCACAC |
| *18S* | ACCGATTGGATGGTTTAGTGAG | CCTACGGAAACCTTGTTACGAC |
